# LeafletAnalyzer, an Automated Software for Quantifying, Comparing and Classifying Blade and Serration Features of Compound Leaves during Development, and among Induced Mutants and Natural Variants in the Legume *Medicago truncatula*

**DOI:** 10.3389/fpls.2017.00915

**Published:** 2017-05-31

**Authors:** Fuqi Liao, Jianling Peng, Rujin Chen

**Affiliations:** ^1^Computing Services Department, Noble Research InstituteArdmore, OK, United States; ^2^Noble Research InstituteArdmore, OK, United States

**Keywords:** LeafletAnalyzer, blade features, serration features, compound leaves, quantification, classification, automation

## Abstract

Diverse leaf forms ranging from simple to compound leaves are found in plants. It is known that the final leaf size and shape vary greatly in response to developmental and environmental changes. However, changes in leaf size and shape have been quantitatively characterized only in a limited number of species. Here, we report development of LeafletAnalyzer, an automated image analysis and classification software to analyze and classify blade and serration characteristics of trifoliate leaves in *Medicago truncatula*. The software processes high quality leaf images in an automated or manual fashion to generate size and shape parameters for both blades and serrations. In addition, it generates spectral components for each leaflets using elliptic Fourier transformation. Reconstruction studies show that the spectral components can be reliably used to rebuild the original leaflet images, with low, and middle and high frequency spectral components corresponding to the outline and serration of leaflets, respectively. The software uses artificial neutral network or *k*-means classification method to classify leaflet groups that are developed either on successive nodes of stems within a genotype or among genotypes such as natural variants and developmental mutants. The automated feature of the software allows analysis of thousands of leaf samples within a short period of time, thus facilitating identification, comparison and classification of leaf groups based on leaflet size, shape and tooth features during leaf development, and among induced mutants and natural variants.

## Introduction

As the primary photosynthetic organs responsible for converting CO_2_ and water to carbohydrate and oxygen in expense of the solar energy, plant leaves not only are indispensable for the growth of plants but also contribute to sustain aerobic lives on earth (Field et al., 1998; Nealson and Conrad, 1999). In nature, plant leaves exhibit tremendous morphological diversities. Leaf diversities are generally described in broad terms such as simple or compound, large or small, narrow or broad, serrated or smooth.

Leaf size and shape are determined by plant developmental programs. As a result, related species and different varieties within a species can be distinguished from each other by their leaf size and shape characteristics. During development, leaves that emerge from successive nodes differ in their size and shape, a phenomenon known as heteroblasty (Tsukaya, 2002; Hunter et al., 2006). Final leaf size and shape are also affected by environmental conditions under which plants are grown. The degree of variation in leaf size and shape in response to the environmental conditions is known as plasticity (Efroni et al., 2010). Because of these, final leaf size and shape reflect morphological adaptations of plants to a particular environment (Tsukaya, 2002) and have been used in the reconstruction of paleoclimate (Peppe et al., 2011). Leaf teeth are projections along the leaf margin present in some plant species. Leaf teeth are sites of vigorous photosynthesis and transpiration in young leaves particularly during early growing season, accelerating growth relative to an equivalent untoothed leaf (Royer and Wilf, 2006). Previous studies have shown that in most regions of the world, the proportion of toothed woody eudicot species scales inversely with the mean annual temperature (MAT) (Peppe et al., 2011; Royer et al., 2012). This relationship has been used as the basis of a technique called leaf margin analysis for reconstructing MAT from fossil plants (Wilf, 1997). Leaf size is also sensitive to climate and has been used to estimate mean annual precipitation (MAP) in a method called leaf area analysis (Wilf et al., 1998).

To facilitate quantitative analyses of leaf size and shape, several analysis software have been reported, including SHAPE (Iwata and Ukai, 2002), LAMINA (Bylesjo et al., 2008), LeafAnalyzer (Weight et al., 2008), LEAFPROCESSOR (Backhaus et al., 2010), and Leaf J (Maloof et al., 2013). However, these programs are designed to analyze simple leaves with simple margins, such as those of *Arabidopsis thaliana* and *Antirrhinum majus*. With these software, users manually or automatically select a small number of reference points on leaf borders and the software then calculates parameters associated with these reference points. Because a limited number of reference points and principle component analysis (PCA) method are used, these software are not designed to quantify precisely leaf shape and margin features such as those of leaf teeth that are important features of leaves in diverse plant species. Recently, several algorithms have been developed to automate leaf tooth analysis (Corney et al., 2012; Jin et al., 2015). However, these algorithms use either triangles as proxies for leaf teeth (Corney et al., 2012) or sparse representation of leaf tooth features (Jin et al., 2015). A computer vision algorithm has been used to learn features from 7,597 chemically cleared leaf samples representing 2,001 genera of botanical families and orders (Wilf et al., 2016).

The objective of this study is to develop a software to automate measurement, analysis and classification of leaflet blade and margin features of compound leaves in *Medicago truncatula*, a model organism for molecular genetic studies of legumes (Fabaceae) (Young et al., 2011). Legumes represents the third largest family of flowering plants, with many economically important crops such as soybean (*Glycine max*), alfalfa (*Medicago sativa*), and many others (O'Brian and Vance, 2007). *M. truncatula* has been selected as a genetic model mainly because (1) it is a diploid species; (2) its genome is relatively small and has been sequenced; and (3) large mutant and ecotype collections are available for functional studies of traits and associated genes (Cook, 1999; Frugoli and Harris, 2001; Choi et al., 2004; Young et al., 2011). *M. truncatula* plants develop trifoliate leaves with leaflets having distinct patterns of serrations at the distal leaflet margin. However, tools for quantitative analysis of blade and margin features of compound leaves in *M. truncatula* have not been developed yet. Here, we describe the development of the software, LeafletAnalyzer, for automatically analyzing leaflet blade and serration features of trifoliate leaves in *M. truncatula*. In contrast to previously-reported software, this software uses all points (pixels) on leaflet borders for measurements and calculation of 54 leaflet size, shape and serration parameters. To use the software, high resolution images of trifoliate leaves of *M. truncatula* plants are first generated using a digital scanner. A build-in filter in the software detects and removes trichomes from the leaf surface. Then, the software detects and generates separate images of three leaflets of a compound leaf. From the leaflet images, it detects and calculates leaf size, shape and serration parameters. A manual software is also available to allow users to make manual adjustments at any steps during the detection and selection processes if errors occur. Elliptic Fourier transformation is also integrated into the software to generate spectral frequency components for each leaflets, which can be used to accurately reconstruct the original leaflet images.

Using this software, we processed and analyzed thousands of leaflet images to quantify leaflet size and shape, and serration characteristics of compound leaves developed on successive nodes on inflorescence stems of wild type *M. truncatula* plants to identify changes in leaflet characteristics during leaf development, and among *M. truncatula* developmental mutants, and *M. truncatula* natural variants. Using an artificial neural network or *k*-means clustering method, we show that different groups of leaflet samples can be statistically classified according to their genotypes or developmental stages. Taken together, this study shows that LeafletAnalyzer can automately and accurately process, measure and classify leaflet samples based on changes in leaflet characteristics to facilitate characterization of compound leaves for functional studies of relevant genes and natural variations.

## Materials and methods

### Plant material and growth conditions

*M. truncatula* wild type (Jemalong A17), leaf shape mutants (all in the A17 background) and natural variants were grown in a growth chamber or a greenhouse with 16-h/8-h day/night light cycle; 22°C/20°C day/night temperature. For leaf shape mutant analysis, the fourth fully-expanded leaves from 2-month-old plants were collected. For wild type and natural variant analyses, leaves from successive nodes on the primary inflorescence stem of 6 weeks-old or 45 days-old plants were collected.

### Generation of high resolution images

Leaves were scanned using an Epson Perfection V700 scanner at a resolution of 800 dpi. Images were saved as TIFF files for subsequent analysis. To avoid shadows along leaf edges and thus increase the accuracy in detection and measurement of leaflets, all leaf samples were scanned against black or gray background. To characterize blade and serration features during leaf development, all six compound leaves developed on successive nodes of a 6 weeks-old wild type plant were detached from the stem, arranged in order, and scanned together to generate a single tiff image. Thirty two plants were used for biological replicates. To compare blade and serration features among four different ecotypes, all seven compound leaves developed on successive nodes of a 45-d-old plant were detached, arranged in order, and scanned as a single tiff image. 20–24 plants were used for biological replicates. To characterize the abnormaty of leaf features in induced mutants, fully expanded compound leaves on the 4th node of nine to twenty 2-month-old plants were detached and scanned to generate multiple tiff images.

### LeafletAnalyzer

LeafletAnalyzer was written in MATLAB (2012–2016) with Image Processing and Visualization toolbox. Thousands of *Medicago truncatula* leaf samples were imaged and analyzed in this study. Original leaf images are deposited into https://dataverse.harvard.edu/dataset.xhtml?persistentId=doi:10.7910/DVN/ZPGVPP; https://dataverse.harvard.edu/dataset.xhtml?persistentId=doi:10.7910/DVN/QLXGBG; and https://dataverse.harvard.edu/dataset.xhtml?persistentId=doi:10.7910/DVN/29PJR1 for public access. Raw data are listed in Supplementary Files 1–3. Screenshots of the operational steps of LeafletAnalyzer are provided in Supplementary File 4.

## Results

### Image processing

**Figure 1:**
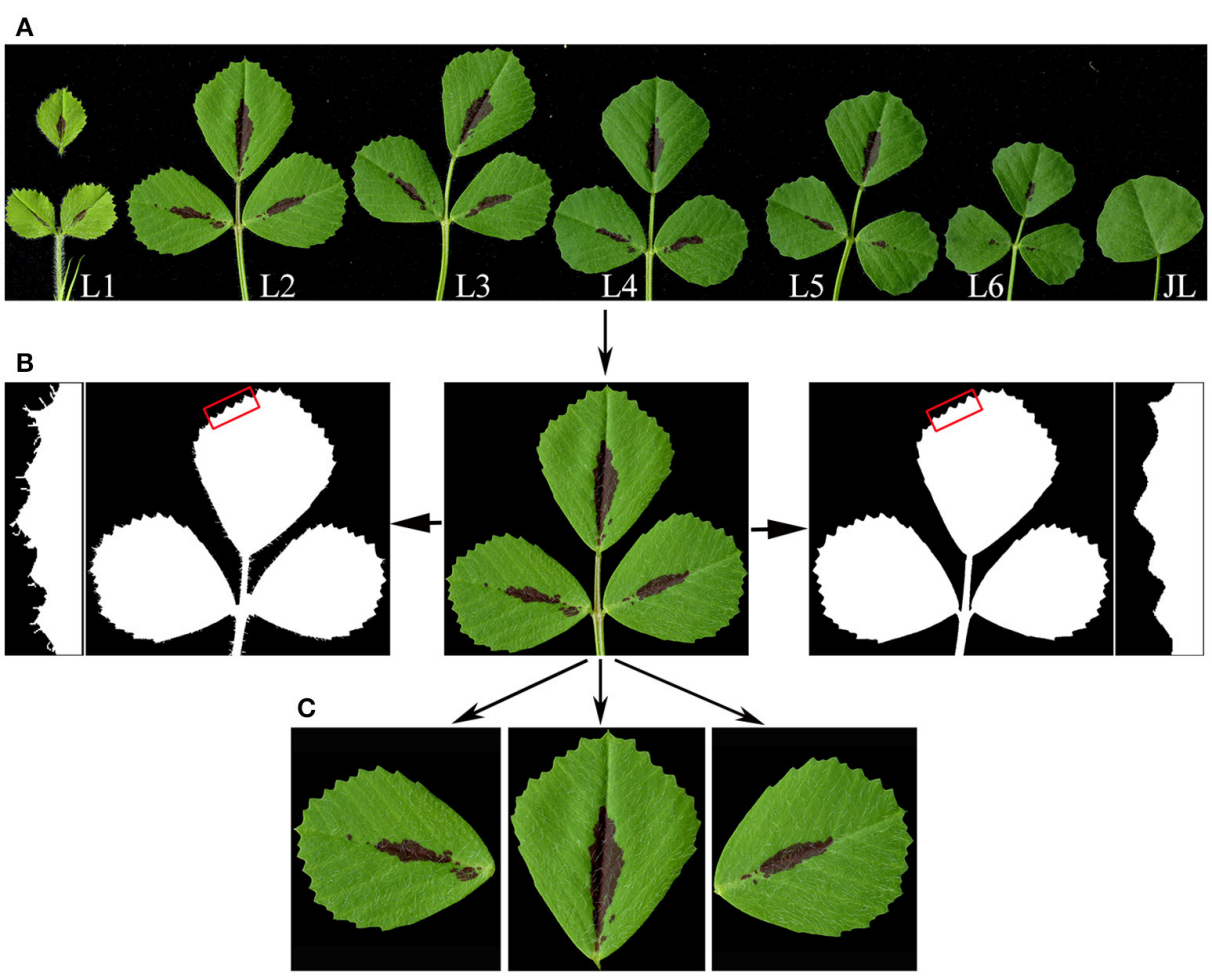
Leaf image processing by LeafletAnalyzer. **(A)** A high resolution image of seven sequentially-ordered leaves, L1 to L6, and the juvenile leaf (JL) of 6-week-old *Medicago truncatula* plants, obtained using a flatbed scanner. Note: the terminal leaflet of L1 was dissected apart to avoid overlapping with lateral leaflets. **(B)** Trichome removal. The original image of a leaf (center) was changed to black and white images, showing before (left panels) and after (right panels) trichome removal. Insets show close-up views of leaflet margins highlighted by rectangles in red. **(C)** Separation of terminal, and left and right lateral leaflets by the software.

Except the first, juvenile leaf, which is simple, all other leaves developed in *M. truncatula* plants are trifoliate (Figure 1A). Because trichomes developed on leaflet margins, if present in a relatively large number, interfere with detection and measurement by LeafletAnalyzer, we implemented an algorithm to automatically detect and remove trichomes from leaf images. Trichomes are distinguished from leaf blades by differences in their color composition. Trichomes are transparent and grayish white in color, whereas leaf blades have mixed green and red colors. The software first converts the original RGB images to the YCbCr color space. By analyzing the blue color distribution, the software detected and removed trichomes from the leaf images (Figure 1B). Alternatively, our elliptic Fourier analysis showed that trichomes were represented as high frequency spectral components and can be removed by a low-pass space filter. The threshold frequency of the filter was determined empirically to remove trichomes but maintain leaflet border features.

Next, leaflets of a trifoliate leaf, one terminal and two lateral, are separated from the rachis and petiole, respectively, to generate three separate leaflet images (Figure 1C). To separate the two lateral leaflets from the petiole, the boundary and centroid (the center-most point) of the leaf are selected from the binary image by the software. Distances between the centroid and each points on the leaflet boundary (margins) are calculated and plotted as a function of their positions (Supplementary Figure 1). Similarly, angles of the centroid to each points on the leaflet margins relative to the horizontal line are also calculated and plotted as a function of positions (Supplementary Figure 1). At each blade and petiole junctions, the calculated distances and angles reached two overlapping points with local bottom or peak values (called turning points), which represent the left and right borders of the blade and petiole junction, respectively (Supplementary Figure 1). Once two adjacent turning points are identified, the software automatically separates the leaflet blade from the petiole and generates two separate leaflet images (Figure 1C; Supplementary Figure 1). After separating the two lateral leaflets from the original leaf image, this process is repeated one more time to separate the terminal leaflet from the rachis (Figure 1C).

Although distances from the centroid to leaflet serration (tooth) tips and sinus (valley) bottoms are also low, their values are much higher than those of the blade and petiole/rachis junctions. By applying a threshold filter, serration tips and sinus bottoms are excluded from the selection. In addition, users can manually select the junction borders if the software did not correctly detect the turning points. This has been seen in some rare cases.

### Detection and calculation of blade length and width

To detect and calculate the leaflet (blade) length, the midpoint of the blade and petiole/rachis junction of a leaflet is selected as the starting point and the distance between the midpoint and the blade margin is calculated as the followings:

$$\begin{array}{l} d_{i}=\;\sqrt{{\left(x_{i}-x_{0}\right)}^{2}+{\left(y_{i}-y_{0}\right)}^{2}} \end{array}$$

where (*x*_0_, *y*_0_) is the midpoint of the blade petiole/rachis junction and (*x*_*i*_, *y*_*i*_) is any point on the blade margin.

The blade length is calculated as the longest distance: L = $\underset{i}{\max}\left\{d_{i}\right\}$

The blade width is calculated as the length of the longest line that is orthogonal to the blade length. To calculate this, a series of orthogonal lines with an interval of five pixels are generated to connect points on the blade margin. The longest line is selected as the blade width and calculated as the followings.

$$\begin{array}{l} w_{i}=\sqrt{{\left(x_{i}-x_{j}\right)}^{2}+{\left(y_{i}-y_{j}\right)}^{2}}\;if\;\frac{\left(x_{i}-x_{j}\right)}{\left(y_{i}-y_{j}\right)}\times \frac{x_{l}-x_{0}}{y_{l}-y_{0}}=1\;\\ \;or-1 \end{array}$$

The blade width is calculated as the longest distance: W = $\underset{i}{\max}\left\{w_{i}\right\}$

where (*x*_*l*_, *y*_*l*_) is any point on the blade length (L), which is used to connect two points on the blade margin (*x*_*i*_, *y*_*i*_) and (*x*_*j*_, *y*_*j*_), in a line orthogonal to the blade length.

**Figure 2:**
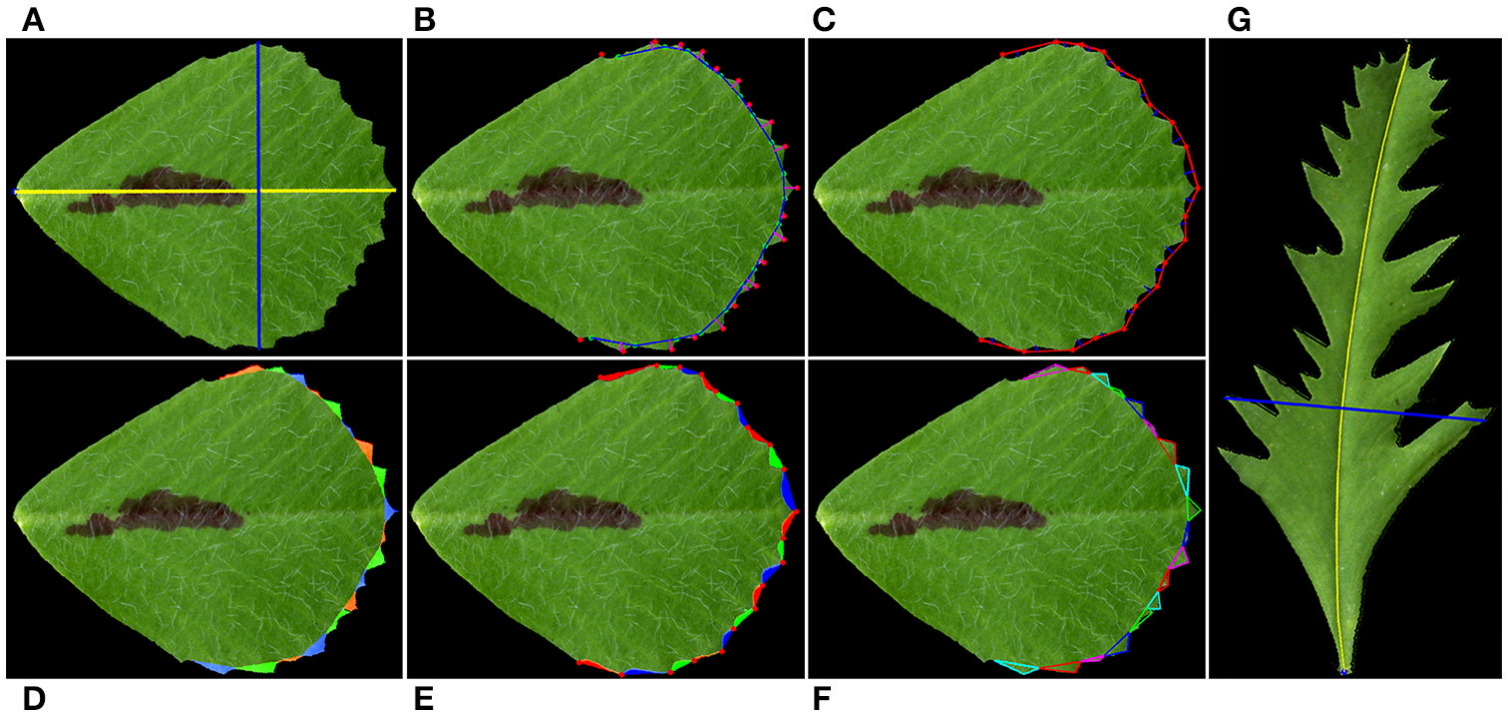
Illustration of leaflet blade and serration parameters measured by the software. **(A)** Blade length (yellow line) and width (blue line). **(B)** Serration (tooth) tips (red dots), sinus (valley) bottoms (cyan dots), tooth base lines (blue lines), and tooth heights (red lines). **(C)** Valley depth (blue lines). Tooth tips are connected by straight lines (red lines). **(D)** Tooth areas (color coded). **(E)** Valley areas (color coded). **(F)** Tooth tip, left and right angles. The software draws a triangle from a tooth tip to two neighboring valley bottoms to estimate the tooth angles. **(G)** The blade length (yellow line) was manually adjusted as a curved line to overlie the midvein of a leaflet of a leaf shape mutant. The blade width (blue line) was automatically selected as the longest line orthogonal to the blade length.

In most cases, the blade length selected by the software overlays perfectly with the midvein (Figure 2A). However, in cases in which leaflets are asymmetric, the selected blade length may deviate from the midvein (Figure 2G). In these cases, end users may manually select three or more points on the midvein, and the software will draw a curve to connect the base and tip of the blade through the selected points and measure the length of the curve as the leaflet length (Figure 2G).

### Detection and calculation of leaflet serrations and sinuses

Leaflet serrations at the distal margin are important features of *M. truncatula* leaves. We observed large variations in leaflet serration characteristics during leaf development in wild type *M. truncatula* plants, developmental mutants and ecotypes (see below). Because this software uses all points (pixels) on the leaflet border, it is designed to detect and calculate accurately leaflet margin parameters (Figures 2B–F).

LeafletAnalyzer detects leaflet serrations (teeth) and sinuses (valleys) as points on the leaflet margin that have local maximal and minimal distances, respectively, to the centroid of the leaflet. The following formula are used to detect the tooth and valley tip (peak) positions.

$$\begin{array}{l} x_{i}>x_{i+1}>x_{i+2}\;and\;x_{i}>x_{i-1}>x_{i-2} \end{array}$$

where *x* is a vector of distances from the centroid to each points on the blade margin. The ith tooth peak position is detected as above. The ith valley peak position is detected as the following:

$$\begin{array}{l} x_{i}<x_{i+1}<x_{i+2}\;and\;x_{i}<x_{i-1}<x_{i-2} \end{array}$$

### Leaf size and shape parameters

**Figure 3:**
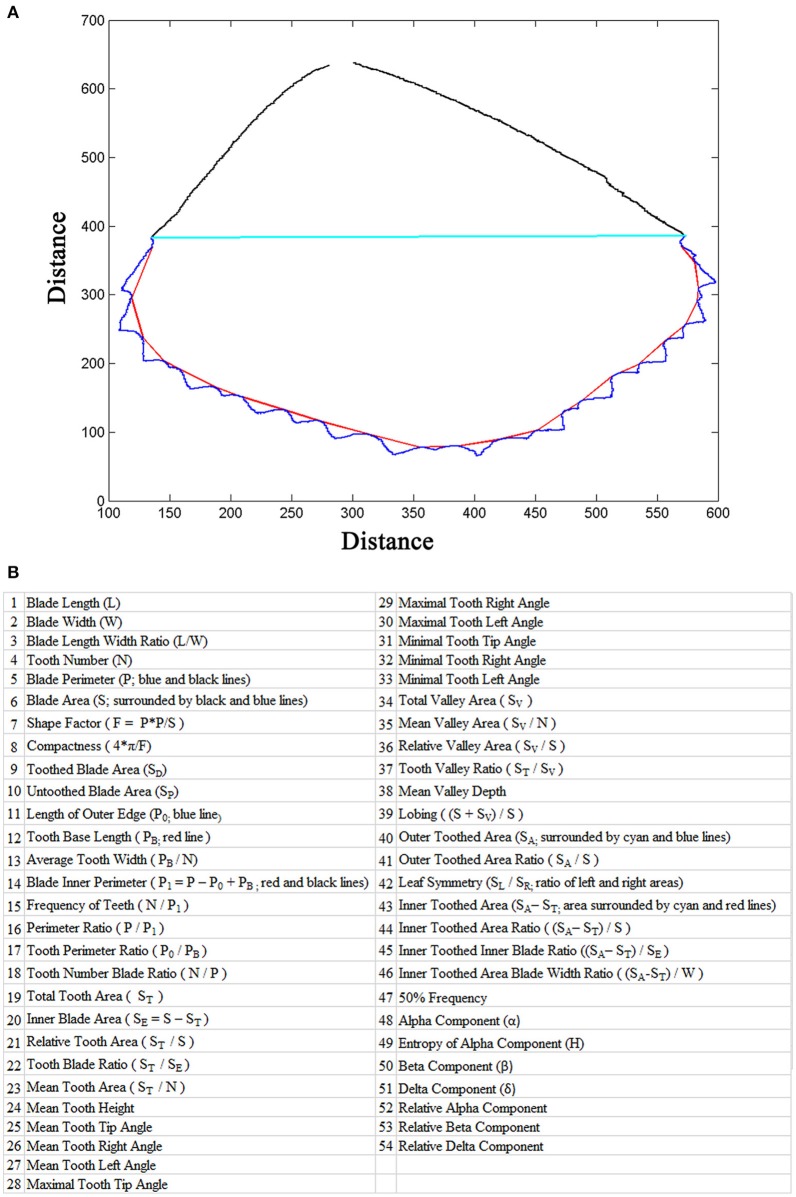
Leaf parameters calculated by the software. **(A)** The outline of a leaflet image generated by the software. The blue line marks the serrations (teeth) and the red line marks the tooth base. The cyan line separates the distal, toothed part and proximal, untoothed part of the blade and the black line marks the margin of the untoothed part of the blade. **(B)** A list of blade and tooth parameters and elliptic Fourier frequency components calculated by the software.

LeafletAnalyzer calculates and exports in excel files more than 50 parameters for each leaflets, including 16 basic leaflet blade and margin parameters, elliptic Fourier frequency component parameters and some derived parameters (Figures 3A,B).

To measure leaflet tooth parameters, a connection line is made between two neighbor valley peak positions. Tooth number is defined as the number of tooth peaks (Figure 2B). Tooth height is the length of the vertical line from the tooth peak position to the connection line (Figure 2B). Tooth area is calculated as the area surrounded by the real tooth border and the connection line (Figure 2D). Tooth perimeter is the total length of the real tooth border and the connection line. To measure tooth angles, including the tooth tip angle, left and right tooth angles, a triangle is made to connect the tooth tip and two neighbor valley peak positions, and the tooth angles are calculated (Figure 2F). Valley parameters are similarly measured as the tooth parameters, except that a connection line is made between two neighbor tooth peak positions (Figures 2C,E).

Additional parameters are calculated in order to comprehensively analyze tooth and leaflet features (Figure 3). The connection lines between two neighboring valley peak positions are combined as the “Tooth Base Line” (Figure 3A; red line), and the length of the tooth base line is defined as the “Tooth Base Length.” A line connecting the starting and ending points of the tooth base line is used to divide the leaflet blade into the top and bottom parts (Figure 3A; cyan line). The total area of the top part is defined as “Outer Toothed Area.” The area surrounded by the tooth base line and the dividing line is defined as “Inner Toothed Area,” which excludes the tooth area (Figure 3A). The total tooth border line is defined as “Length of Outer Edge” (Figure 3A). The inner toothed area and the bottom part of the leaflet blade are combined as “Inner Blade Area,” and the border of the inner blade area is defined as “Blade Inner Perimeter” (Figure 3A). The leaflet length is used to divide the leaflet blade into the left and right parts. The ratio of the left and right blade area is defined as “Leaflet Symmetry” (Figure 3). “Tooth Base Length” and “Inner Toothed Area” are used to describe the degree of tooth distribution. “Inner Toothed Area Ratio” shows the proportion of the toothed area in total leaflet area (Supplementary Figure 3). “Tooth Number Blade Ratio” indicates the density of teeth relative to the leaflet area. Figure 3B lists the parameters and their definitions used in this study. In contrast to previously-published software, LeafletAnalyzer uses the real shape of teeth to describe serration features.

### Elliptic fourier description

The elliptic Fourier function is integrated into the software for leaflet shape and size analyses. Directional variations in leaflet blades and margins are converted into an one-dimensional array, and applied to elliptic Fourier transformation (Neto et al., 2006). In total, 120 frequency components of both amplitudes and phases are calculated. The frequency components are normalized to be invariant with rotation, translation and the starting point. Therefore, the elliptic Fourier description is only related to the shape and size of the leaflet blade and margin.

In this study, we identified that the amplitude components of the elliptic Fourier translation are sufficient for the description of the blade and margin features. We combined the amplitude components along the x and y directions as the following:

$$\begin{array}{l} A{\left(f\right)}^{2}=A{\left(f\right)}_{x}^{2}+A{\left(f\right)}_{y}^{2} \end{array}$$

where f is the frequency, *A*(*f*)_*x*_ and *A*(*f*)_*y*_ are the power spectral A(f) projected in the x and y direction, respectively.

Out of the 120 spectral components, the low frequency components (1–19th) are defined as “Delta” components, whereas the middle frequency components (20–55th) and the high frequency components (56–120th) are defined as “Alpha” and “Beta” components, respectively. “50% Frequency” is defined as the frequency at which the sum of the total power spectral A(f) is divided into two equal halves.

The elliptic Fourier description provides a method to analyze blade and serration characteristics in frequency zones. Firstly, elliptic Fourier analysis allows reconstruction of the actual blade and serration using frequency components. By analyzing more than thousands of different groups of *M. truncatula* leaflets samples, we show that Delta components mainly correspond to the blade outline, and Alpha components to leaflet teeth characteristics. Secondly, elliptic Fourier description reflects integrated features of blade and serration in a leaflet. For an example, when a leaflet contains different sizes of teeth, especially when small serrations are less than 10% of large serrations, the average tooth height and area will not accurately reflect features of all teeth. Thus, the frequency components of the elliptic Fourier description become important parameters to reflect the variation of tooth features. The frequency components of elliptic Fourier description have been successfully used in the classification of different leaflet groups in this study.

### Statistical analysis

Statistical analysis included in LeafletAnalyzer uses the statistical toolbox of MATLAB. Because most of the blade and margin parameters are not in normal distribution, comparisons of multiple leaf groups are performed using both one way ANOVA and Kruskal-Wallis tests. Data are represented by mean ± s.e. Significant differences analysis is made based on 95% CI (confidence interval). Standard deviation/mean was used to evaluate data variations. A majority of leaflet groups has leaflet blade and serration parameters within mean ± 0.2 standard deviation/mean.

### Classification of leaflet groups

**Figure 4:**
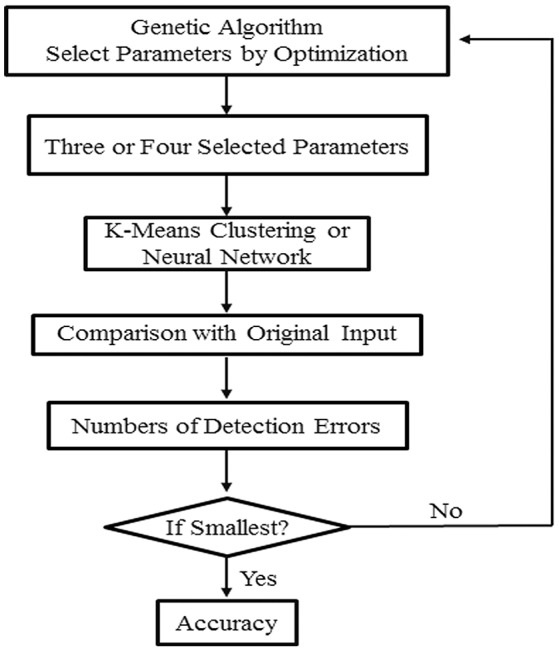
A flow chart of classification of leaflet samples using *k*-means clustering or artificial neural network method. A combination of three to four leaflet blade and serration parameters were selected by the genetic algorithm to classify leaflet samples with the highest accuracy. This process is reiterated multiple times to generate a list of parameter sets that can be used to classify leaflet samples with the highest accuracy.

Classification of four or more leaflet groups are made by using the *k*-means or artificial neural network method. Three or four parameters are selected as a group input to the *k*-means or artificial neural network programs for classification (Figure 4). The accuracy of classification is defined as the percentage of correct classifications. Combinations of three or four parameters that give rise to at least 85% accuracy are selected.

For classification with artificial neural network (Kim, 2010; Saravanana and Sasithra, 2014; Aboukarima et al., 2015), a feedforward neural network with 3–15 hidden layers is used. All leaf samples are mixed together at first. 75% of the leaf samples are used in training, and the remaining 25% are used in identification. This process is repeated almost 30 times, each time with a different set of training samples. We used a genetic algorithm to facilitate the optimal selection of three or four parameters (Figure 4).

### Characterization of blade and serration features during leaf development in *M. truncatula*

Previously, we have shown that the proximodistal axis development varies in leaves developed on different nodes (Wang et al., 2008; Peng et al., 2011; Ge et al., 2014). Visual examination suggests that leaf shape and size are also variable at different developmental stages (Figure 1A). To quantify differences in shape and size during leaf development, we measured and analyzed blade and serration parameters of trifoliate leaves developed on successive nodes of 6 weeks-old wild type *M. truncatula* plants, using LeafletAnalyzer. Thirty two biological replicates were used for statistical analyses. For each replicate, all six trifoliate leaves, labeled sequentially by the software as L1 (the youngest measurable leaf from the shoot apex) to L6 (the oldest leaf from the shoot apex), as well as the juvenile leaf (the first leaf emerged on the stem) were included in the original images, although the juvenile leaf was excluded from further analysis (Figure 1A).

**Figure 5:**
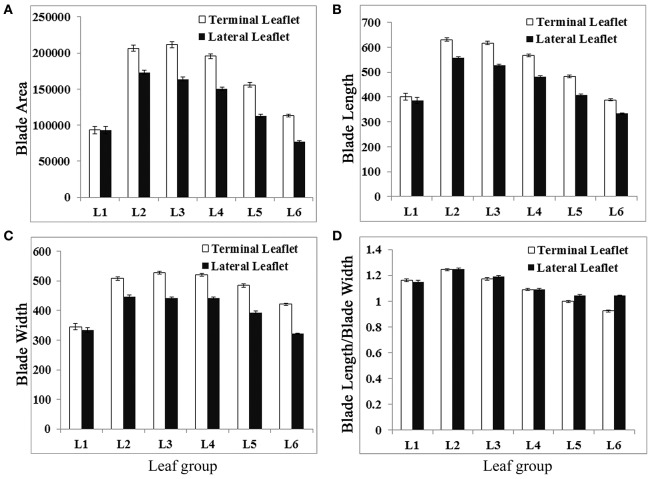
Measurements of leaflet blade parameters of L1 to L6 in wild-type *M. truncatula* cv. Jemalong A17 plants. Measurements of blade area **(A)**, blade length **(B)**, blade width **(C)**, and blade length width ratio **(D)** from L1 to L6, leaves collected sequentially from the shoot apices of 6-week-old plants. Shown are means ± s.e., *n* = 32.

Analysis results indicate that terminal and lateral leaflets in L1 were similar in size and shape (Figure 5). From L2 to L6, there were significant differences in size and shape between terminal and lateral leaflets with 95% CI (Figure 5). Terminal leaflets were always larger than lateral leaflets (Figure 5A). Interestingly, terminal leaflets were the largest in L3, and lateral leaflets were the largest in L2 (Figure 5A). The leaflet length was the longest for both terminal and lateral leaflets in L2, and reduced gradually from L3 to L6 (Figure 5B). However, terminal leaflets were always longer than lateral leaflets and the differences were statistically significant with 95% CI. The width of leaflets changed very little from L2 to L4, but reduced in L5 and L6, and the differences were significant with 95% CI between terminal leaflets and lateral leaflets (Figure 5C). The analysis results also indicate that lateral leaflets reduced width more than terminal leaflet in L5 and L6 (Figure 5C). The blade length width ratio was significantly different between terminal and lateral leaflets with 95% CI only in L5 and L6. The blade length width ratio was less than 1 only in terminal leaflets in L6, indicating that only terminal leaflets were wider in shape in L6 (Figure 5D).

**Figure 6:**
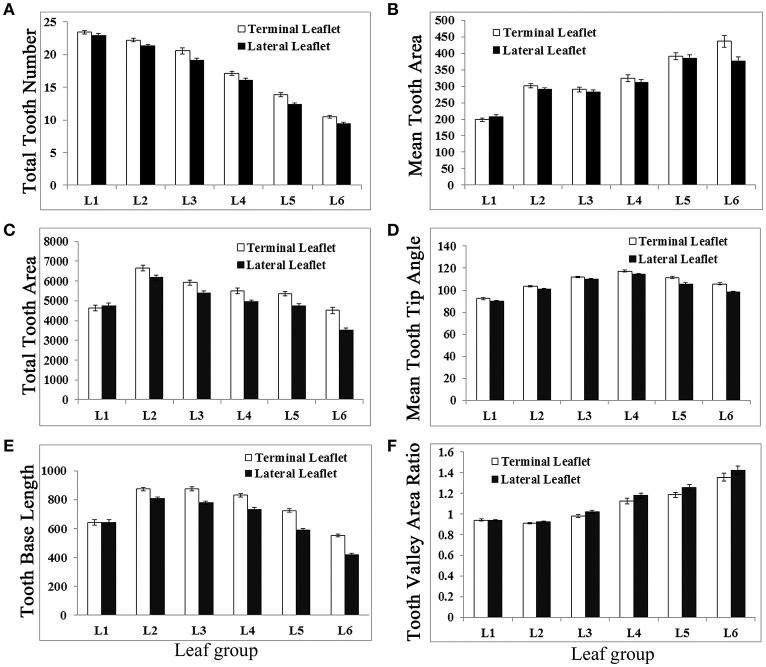
**Measurements of leaflet serration parameters of L1 to L6 of *M. truncatula* cv. Jemalong A17 plants**. Measurements of total tooth number **(A)**, mean tooth area **(B)**, total tooth area **(C)**, mean tooth tip angle **(D)**, tooth base length **(E)**, and tooth valley area ratio **(F)** from leaf 1 (L1) L6 of 6-week-old *M. truncatula* plants. Shown are means ± s.e., *n* = 32.

Analysis results show that newly emerged leaves had the highest number of serrations (teeth) at the distal margin, even though they had the smallest blade area (Figure 6A). From L1 to L6, total serration number was gradually decreased in a nearly linear fashion in both terminal and lateral leaflets (Figure 6A). In L1, terminal and lateral leaflets had a similar number of teeth; but from L2 to L6, terminal leaflets always had more serrations than lateral leaflets (Figure 6A). The average tooth area increased in general from L1 to L6 (Figure 6B). Interestingly, the total tooth area was increased from L1 to L2 but gradually decreased from L2 to L6 (Figure 6C). The average tooth tip angle was gradually increased from L1 to L4 and decreased from L4 to L6 (Figure 6D). The “tooth base length” was increased from L1 to L2 but decreased gradually from L2 to L6 (Figure 6E). On the other hand, the “tooth valley area ratio” remained similarly low in L1 and L2 but increased steadily from L3 to L6 (Figure 6F). Except L1, terminal leaflets in general had significantly (95% CI) higher “tooth number,” larger “total teeth area,” and longer “tooth base length” but smaller “tooth valley area ratio” than lateral leaflets (Figure 6). The average “tooth tip angle” was significantly different between terminal and lateral leaflet only in L5 and L6. Because the “total teeth area” and “tooth base length” shared a similar trend of changes with leaflet length and leaflet area during leaf development, a large leaflet tended to have larger teeth and a longer “tooth base length.” In summary, LeafletAnalyzer facilitated quantitative analyses of leaflet size and shape and enabled identification of directions and degrees of changes in leaflet size and shape during leaf development in *M. truncatula*.

**Figure 7:**
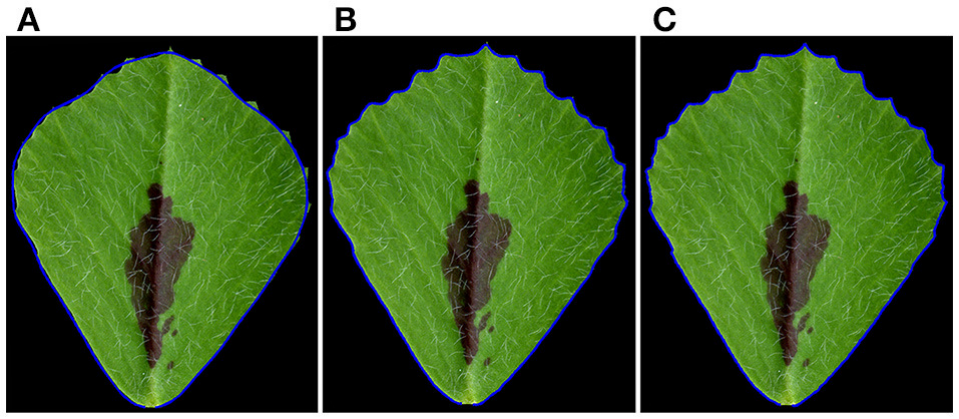
Reconstruction of leaflet shapes using spectral frequency components of the elliptic Fourier analysis. **(A)** Reconstruction of the outline of the original leaflet, using Delta components (1st–19th spectral components) of the elliptic Fourier analysis. Note: only the outline of the leaflet was reconstructed. **(B)** Reconstruction of the blade and serration features using both Delta) and Alpha components (1st–55th spectral components) of the elliptic Fourier analysis. Note: most of the blade and tooth features were reconstructed. **(C)** Reconstruction of the blade and serration features using Delta, Alpha and Beta components (1st–120th spectral components). Note: all of the blade and tooth features were reconstructed. Shown are overlays of the original leaflet with the reconstructed leaflet outlined by a blue line.

Using elliptic Fourier analysis, we evaluated the distribution of a total of 120 spectral components of leaflet parameters from L1 to L6 in 6 weeks-old plants. The original leaflet shape can be precisely reconstructed using the 120 frequency components of the elliptic Fourier transformation. To answer how the spectral components of the elliptic Fourier transformation related to leaflet shape, we reconstruct the leaflet shape by an inverse elliptic Fourier transformation with a subset of the spectral components. When the Delta components were used, only the outlines, but not the margin features, of the original leaflets were reconstructed (Figure 7A). When Delta and Alpha components were used, almost all of the blade and margin features were reconstructed (Figure 7B). Thus, the Alpha components are mostly related to the margin features. When all 120 frequency components were used, the blade and margin features were completely reconstructed (Figure 7C). Thus, the “Beta” components are related to small, local variations of the blade margin.

**Figure 8:**
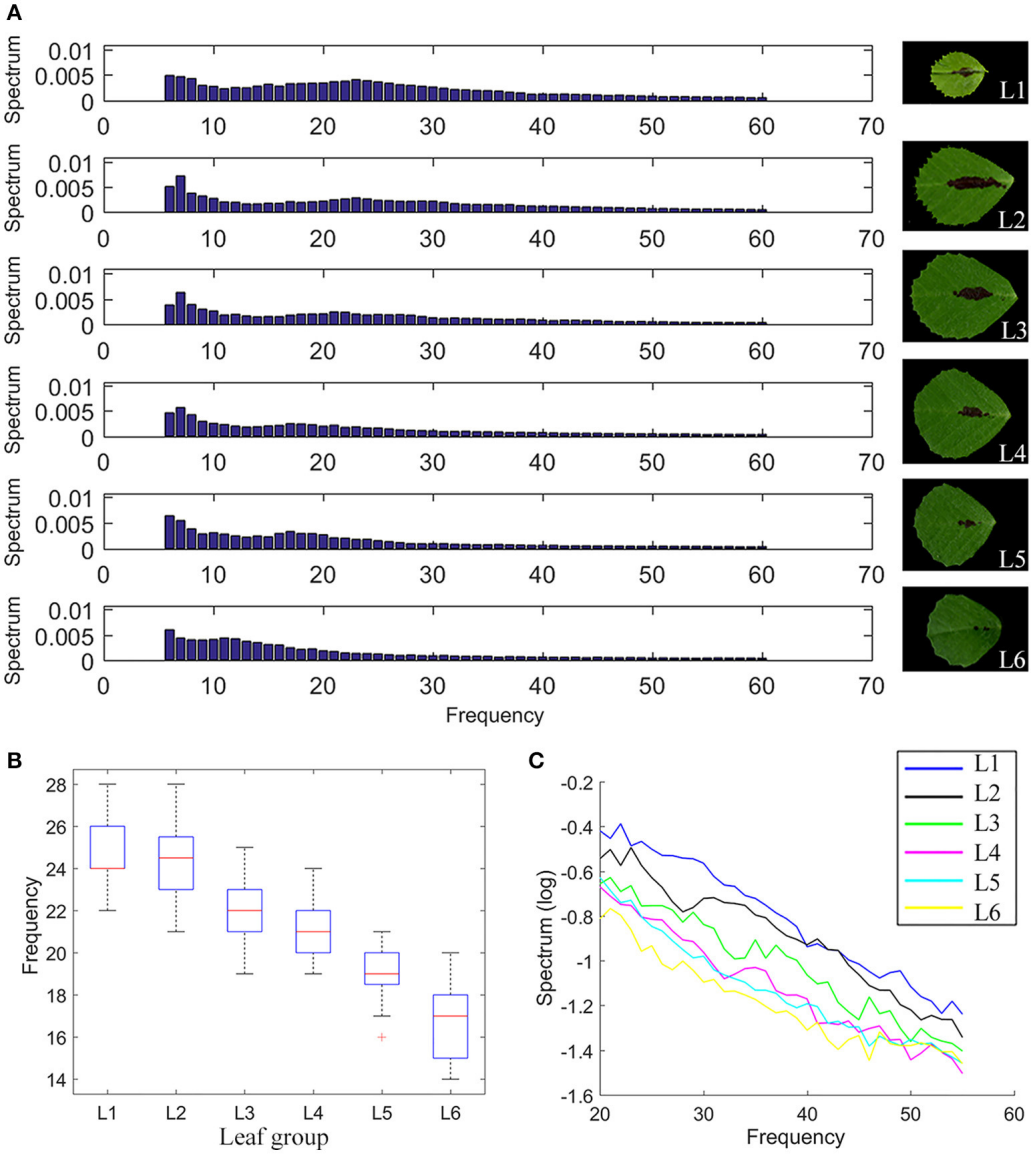
Elliptic Fourier analysis of *M. truncatula* wild-type leaves. **(A)** Intensity distribution of the spectral frequency components of the elliptic Fourier analysis of compound leaf 1 (L1) to compound leaf 6 (L6) in 6-week-old *M. truncatula* cv. Jemalong A17 plants, showing a gradual shift from high frequency to low frequency components from L1 to L6. Right panels show representative images of corresponding leaflets. **(B)** A boxplot of 50% frequency of L1–L6. Red lines denote the median values, upper and lower edges of boxes denote 75 and 25% percentiles, respectively, whiskers denote the most extreme data points that are not considered outliers, and outliers are marked individually, *n* = 32. **(C)** Decrease of Alpha components (20th–60th spectral components) from L1 to L6.

Analysis results show that “50% frequency” and “Alpha components” decreased continually from L1 to L6 (Figures 8A–C), supporting the observation that the tooth number and tooth height decreased but the “tooth valley area ratio” increased from L1 to L6 (Figure 6F).

Because the direction and degree of changes in leaflet blade and margin parameters are in most cases statistically significant among leaflet groups from L1 to L6, we tested whether these parameters can be used to classify leaflet groups. We used an artificial neural network to classify leaflet groups from L1 to L6, using a combination of three or more blade and margin parameters. Supplementary Table 1 lists ten combinations of three parameters that gave rise to the classification accuracy ranging from 90.4 to 97.9% (Supplementary Table 1). Consistent with the statistical analysis results, both the blade and margin parameters are important for the classification. The classification results suggest that the blade and margin features are statistically distinct in leaflet groups from L1 to L6 and these can be used successfully to classify leaflet groups during leaf development in *M. truncatula*.

### Quantitative analysis and classification of blade and margin features of *M. truncatula* natural variants

**Figure 9:**
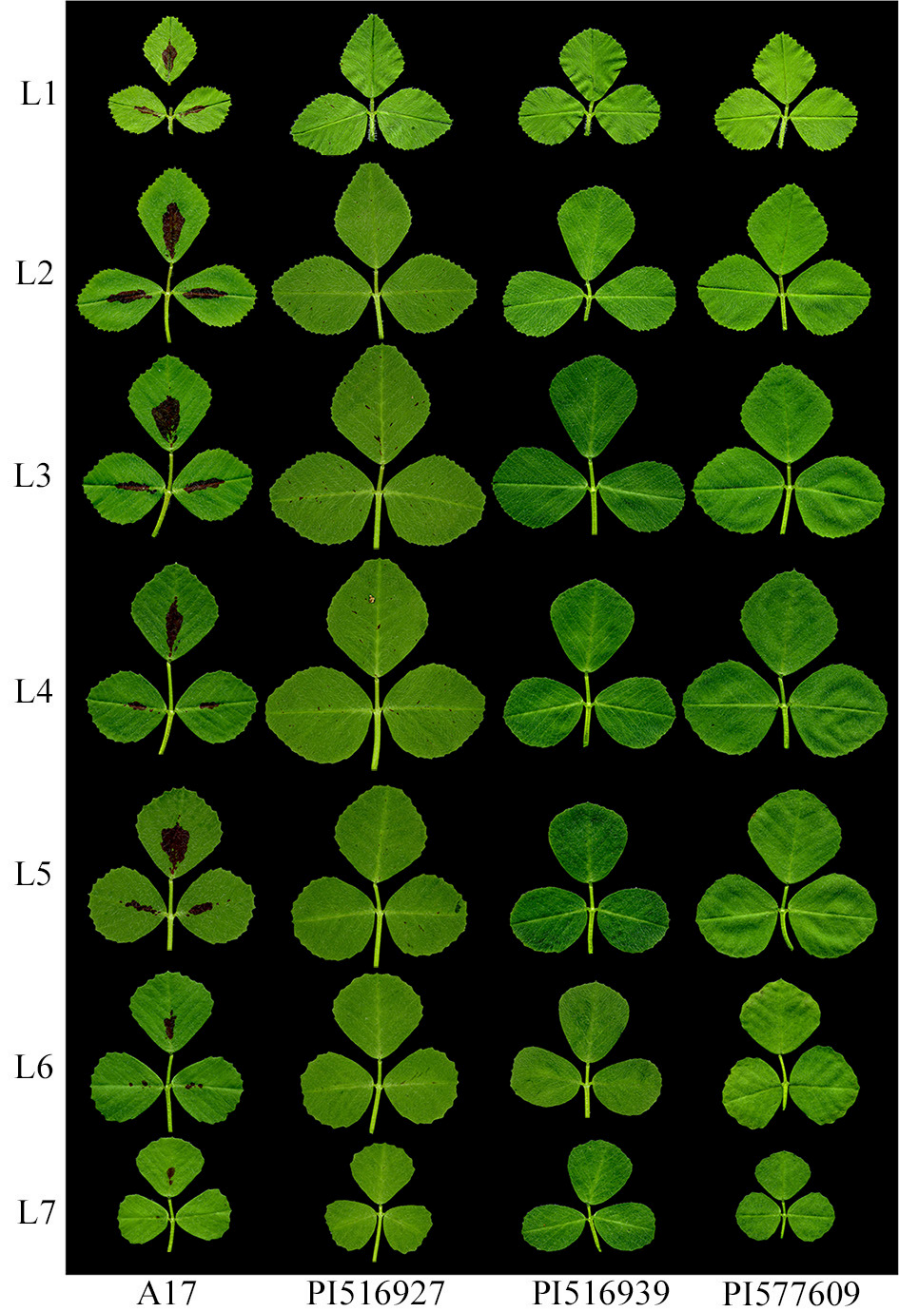
Representative images of leaves of *M. truncatula* cv. Jemalong A17 and natural variants PI516927, PI517939 and PI577609. L1 to L7 denote leaves collected sequentially from the shoot apices of 7-week-old plants.

Using LeafletAnalyzer, we calculated and compared blade and margin parameters of trifoliate leaves of three *M. truncatula* natural variants (PI516927, PI516939, and PI577609) and the reference plant, Jemalong A17. For this, seven to 20 biological replicates were used. For each replicate, seven trifoliate leaves were collected from successive nodes on the stem of 45 days-old plants (Figure 9).

**Figure 10:**
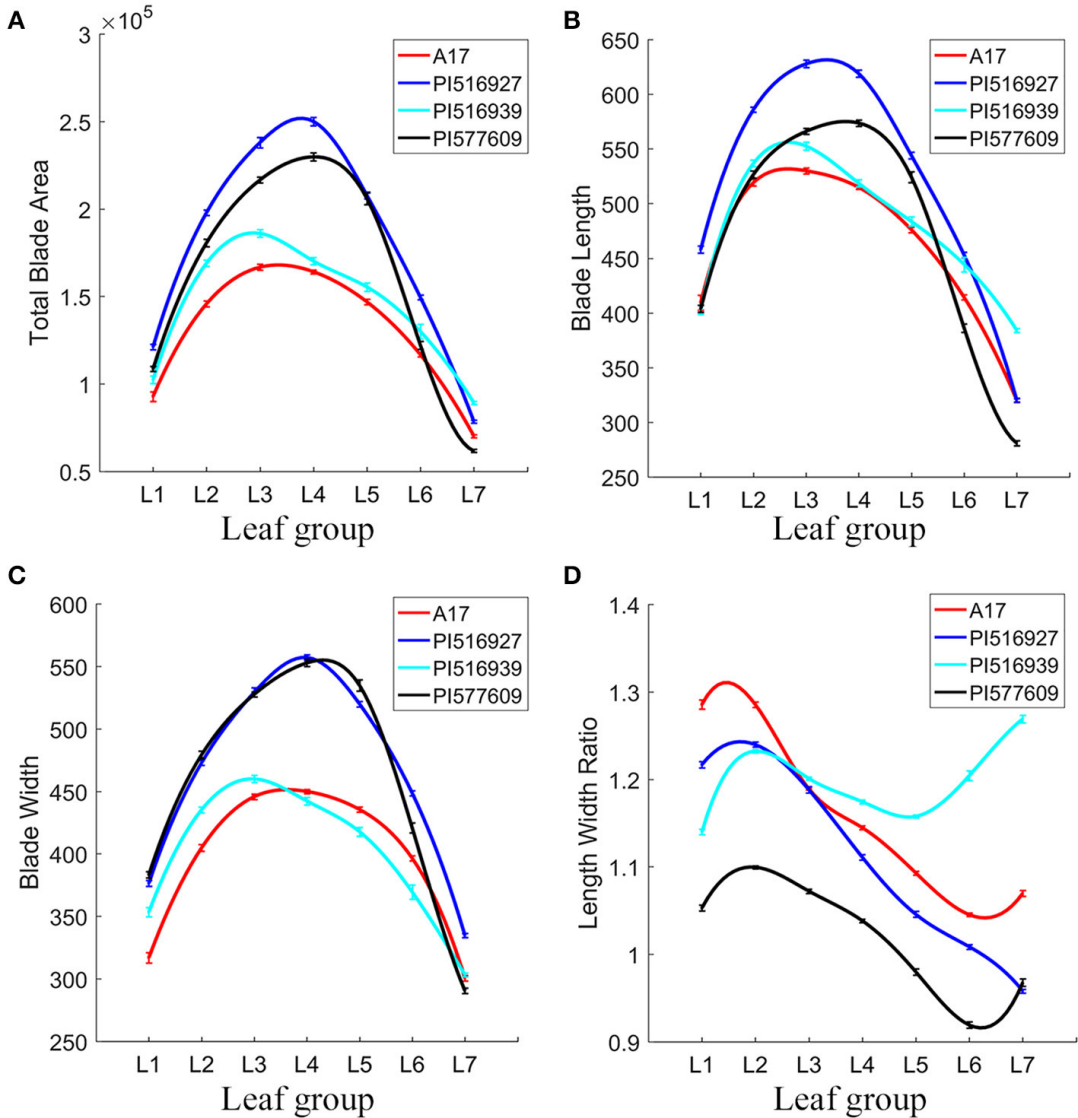
Analysis of leaflet blade features of leaves in *M. truncatula* natural variants. To fit the variance of the average of blade parameters among seven leaf groups (L1–L7), cubic spline curve fitting was used. Shown are total blade area **(A)**, blade length **(B)**, blade width **(C)**, and blade length width ratio **(D)** of L1–L7 in 7-week-old plants. *M. truncatula* cv. Jemalong A17, PI516927, PI516939, and PI577609 plants were shown in different colors.

Overall, we observed significant differences in blade and margin parameters as well as the degree of changes from L1 to L7 among the natural variants and the reference plants (Figure 10). For examples, from L1 to L7, PI516927 had the largest blade size, and Jemalong A17 and PI516939 had the smallest blade size (Figure 10A). In a quantitative term, compared with PI516927 (normalized as 1), the area of lateral leaflets ranged between 0.656 and 0.787 in Jemalong A17 and 0.681–0.854 in PI516939 (Figure 10A). From L1 to L6, PI577609 had the second largest blade size. Interestingly, in L7, PI516939 had the largest blade size, PI577609 had the smallest blade area and the other two had similar, intermediate blade size (Figure 10A).

In L1 and L2, Jemalong A17, PI577609 and PI516939 had similar blade length (Figure 10B). From L1 to L6, PI516927 had the longest blade length (Figure 10B). From L3 to L5, PI577609 and PI516939 had the second and third longest blade length and Jemalong A17 had the shortest blade length (Figure 10B). However, in L6 and L7, PI577609 had the shortest blade length, and, in L7, PI516939 had the longest blade length (Figure 10B). From L1 to L6, both PI516927 and PI577609 had the longest blade width (Figure 10C). From L1 to L3, Jemalong A17 had the shortest blade width (Figure 10C). From L4 to L6, PI516939 had the shortest blade width (Figure 10C). In L6 and L7, the longest blade width was found in PI516927 (Figure 10C).

By combining the length and width information, the blade length width ratio represents an important shape parameter. From L2 to L6, the blade length width ratio was in general decreasing in all four genotypes, with an exception that PI516939 had increased blade length width ratios in L6 and L7 (Figure 10D). These results indicate that in general leaflets in newly developed leaves grew preferentially along the proximodistal axis; whereas leaflets in older leaves grew preferentially along the mediolateral axis in these genotypes. However, there were some exceptions. For examples, the blade length width ratio was increased from L1 to L2 in PI516939, from L5 to L7 in PI516939, and in L6 and L7 in Jemalong A17, and the increase only occurred in lateral leaflets (Figure 10D). From L2 to L7, the blade length width ratio decreased almost linearly in PI516927 (Figure 10D).

From L1 to L7, PI577609 had the lowest blade length width ratio (Figure 10D). On the other hand, in L1 and L2, Jemalong A17 had the highest ratio, followed by PI516927 and PI516939. From L3 to L7, PI516939 had the highest ratio, followed by Jemalong A17 and PI516927 (Figure 10D). These results indicate that the degree of changes along the leaf proximodistal and mediolateral axes (reflecting compactness) varies among these genotypes during development.

**Figure 11:**
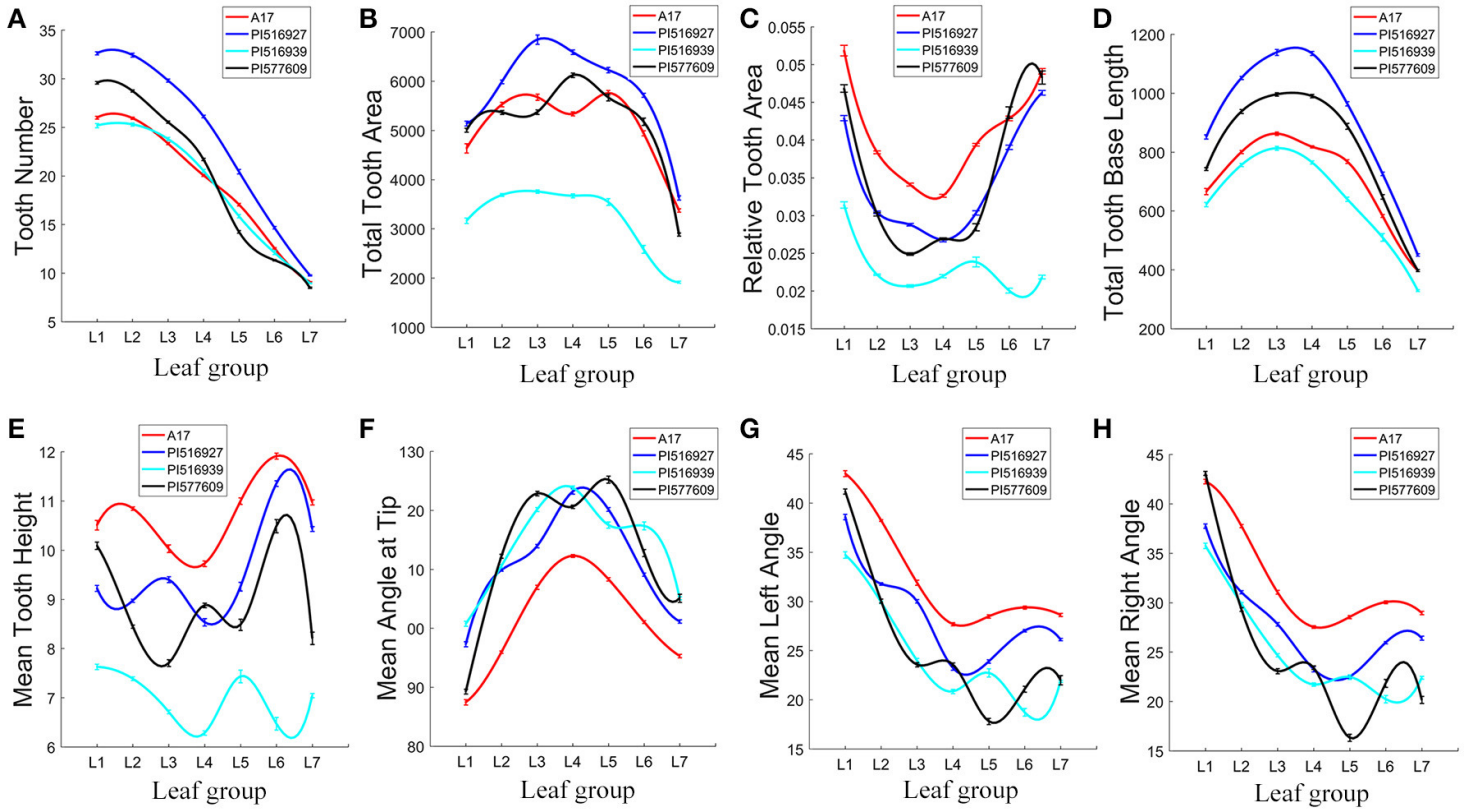
Analysis of leaflet serration features of leaves in *M. truncatula* natural variants. To fit the variance of average of serration parameters among seven leaf groups (L1–L7), cubic spline curve fitting was used. Shown are tooth number **(A)**, total tooth area **(B)**, relative tooth area **(C)**, total tooth base length **(D)**, mean tooth height **(E)**, mean angle at tip **(F)**, mean left angle **(G)**, and mean right angle **(H)** of L1–L7 in 7-week-old plants. *M. truncatula* cv. Jemalong A17, PI516927, PI516939, and PI577609 plants are shown in different colors.

The total tooth number was either similar in L1 and L2 or slightly reduced in L2 in these four genotypes. From L2 to L7, the tooth number was almost linearly reduced in all four genotypes (Figure 11A). Compared with other genotypes, PI516927 had the highest tooth number from L1 to L6 (Figure 11A). From L1 to L4, PI577609 had the second highest number of teeth (Figure 11A). However, in L5 and L6, the tooth number was the lowest in PI577609 (Figure 11A). Jemalong A17 and PI516939 had similar tooth number from L1 to L7 (Figure 11A). In L7, the number of teeth was very similar among Jemalong A17, PI516927 and PI516939 (Figure 11A). Compared with PI516927, the tooth number from L1 to L6 was reduced by 12–27% in the other three genotypes (Figure 11A).

From L1 to L7, the total tooth area was the highest in PI516927 and lowest in PI516939 (37.4% to 65.7% of that of PI516927), except that PI516927 and PI577609 were similar in the tooth area in L1 (Figure 11B). Jemalong A17 and PI577609 had intermediate tooth area in most of the leaflet groups (79.5–95.4% of that of PI516927), except L1 (Figure 11B).

The relative tooth area (total tooth area/blade area) was the highest in Jemalong A17 (116.9–130.7% of that of PI516927) from L1 to L5) and lowest in PI516939 from L1 to L7, except that PI577609 had similar relative tooth areas as Jemalong A17 in L6 and L7 (Figure 11C). From L1 to L7, the relative tooth areas were intermediate in PI516927 and PI577609, except that for PI577609 in L6 and L7 (Figure 11C).

The following parameters were used to further compare the size and shape of serrations: tip, left and right angles, and height and baseline length of teeth (Figures 11D–H). Jemalong A17 had the shortest tooth baseline (Figure 11D), the largest tooth height (106.7–123.1%) (Figure 11E), and the smallest tooth tip angle (84.9–94% compared with that of PI516927) from L1 to L7 (Figure 11F), indicating that leaflet serrations were taller and sharper from L1 to L7 in Jemalong A17 than other genotypes. On the other hand, PI516939 had the smallest tooth height, ranging from 54.7 to 82.7%, and the tooth area, ranging from 52.6 to 80.8% of that of PI516927 (Figures 11B,E).

**Figure 12:**
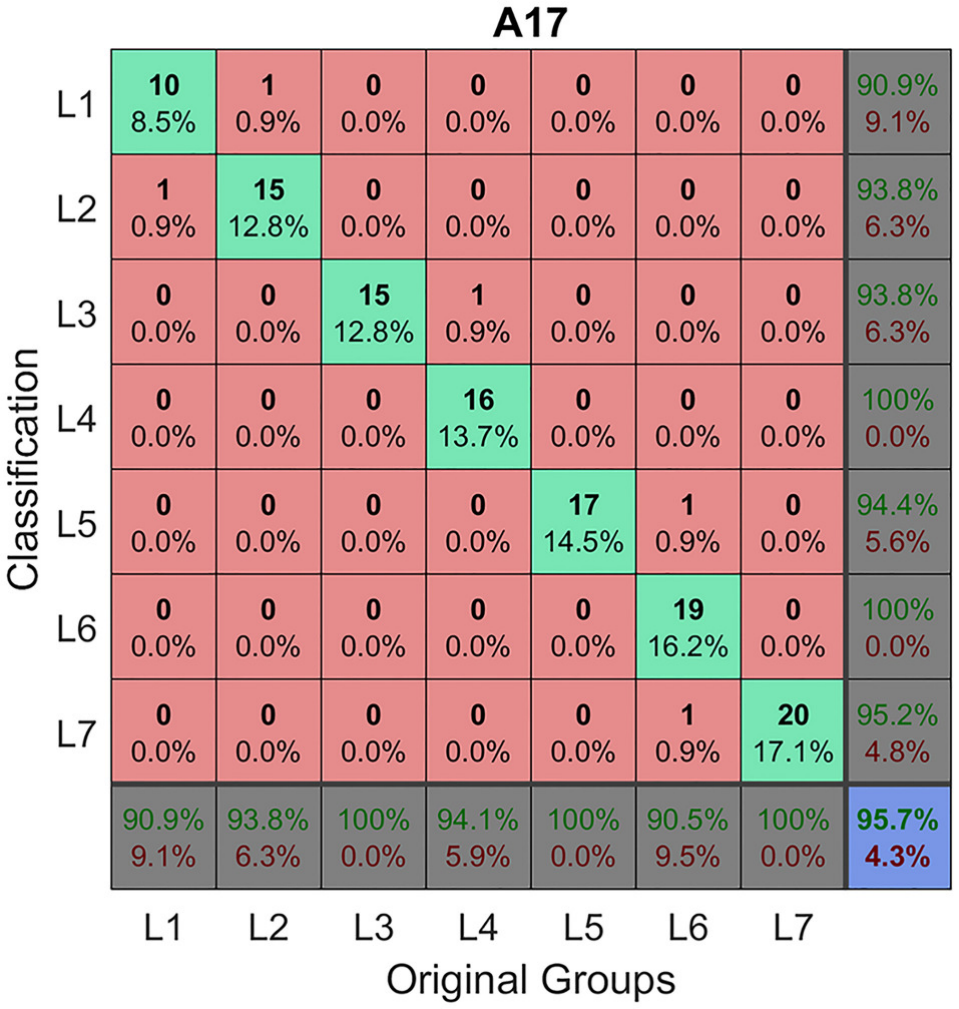
Classification of leaf groups of *M. truncatula* wild type plants. An artificial neural network method was used to classify leaf groups L1–L7 of 45 days-old *M. truncatula* cv. Jemalong A17 plants, using three calculated parameters, total tooth number, blade length, and blade length width ratio. Shown is a summary of the classification results. The classification accuracy was calculated as 95.7%. X-axis denotes original leaf groups as the input; Y-axis denotes leaf groups after classification. Numbers in the pink cells represent the number of leaflet samples that were misclassified; whereas numbers in the green, gray and purple cells represent the number and percentage of leaflet samples that were correctly classified.

Because of statistically significant changes in blade and serration parameters seen among the leaf groups within and among genotypes (Figures 10, 11), we tested whether these parameters can be used to classify leaf groups within or among genotypes, using an artificial neural network method as described earlier (Figure 4). The analysis results show that up to 32 sets of three blade and serration parameters can be used to classify leaf groups within or among the four genotypes with at least 90% of accuracy. The classification was based on parameters of left or right lateral leaflets, terminal leaflets, or leaflets as a group. Supplementary Tables 2, 3 listed ten combinations of parameters for each analysis. Figure 12 shows an example of the classification results for seven leaflet groups, L1 to L7 in 45-days-old wild type *M. truncatula* (A17) plants. The classification accuracy was calculated as 95.7% in this case.

**Figure 13:**
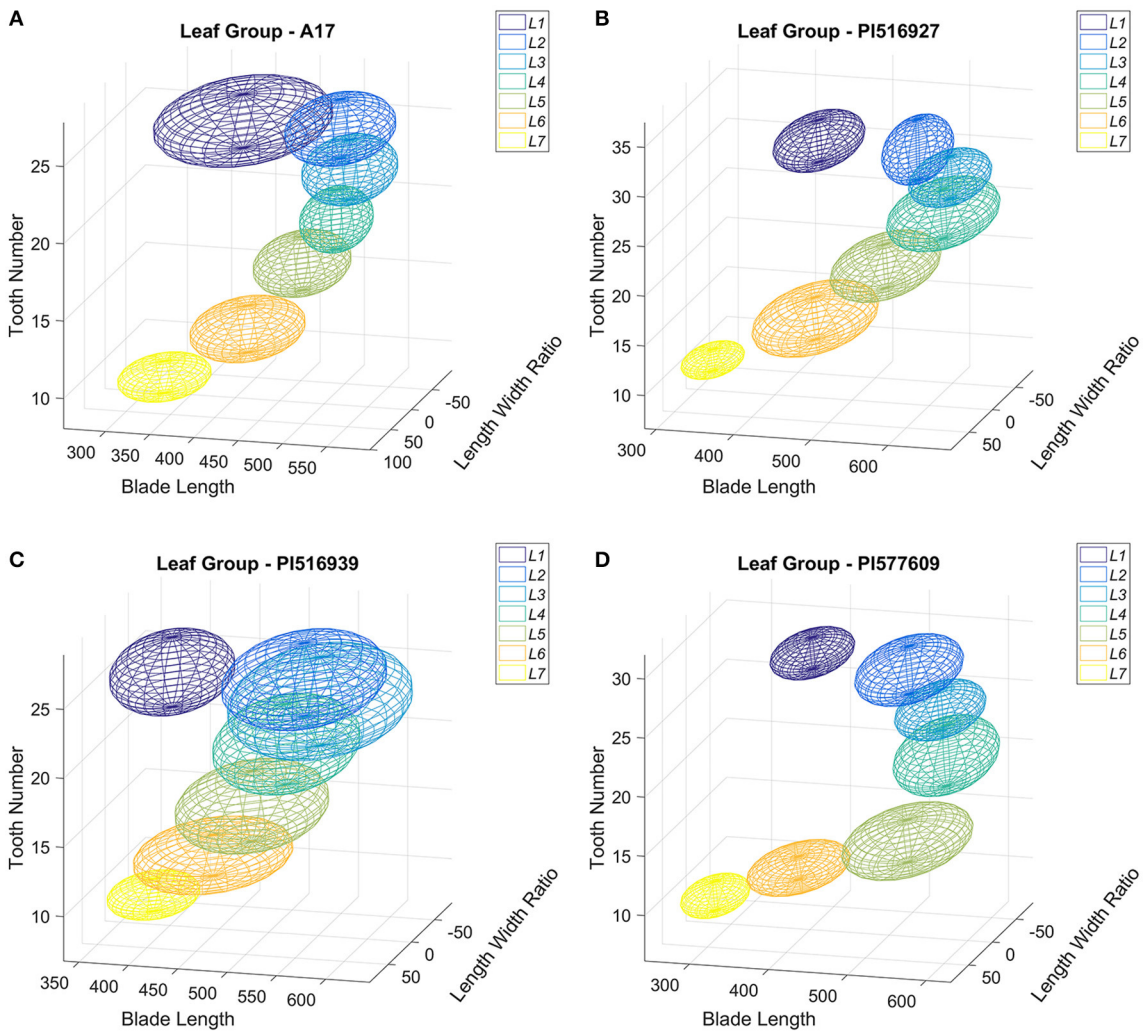
Distribution in three-dimensional morphospace of leaf groups of *M. truncatula* wild type and natural variants plants. Distribution of leaf groups L1–L7 of 45-days-old *M. truncatula* wild type, A17 **(A)** and natural variants, PI516927 **(B)**, PI516939 **(C)**, and PI577609 **(D)** plants in three-dimensional morphospace defined by total tooth number, blade length and blade length width ratio. Each leaf group is represented by an ellipsoid with 95% CI.

Next, we visualized the distribution of leaf groups (L1–L7) in a three-dimensional morphospace confined by three blade and serration parameters selected from the classification experiments. Figure 13A shows that the seven leaf groups, L1–L7 of *M. truncatula* cv. Jemalong A17 plants, occupied distinct space in the 3D morphospace confined by tooth number, blade length and length width ratio. Similarly, the seven leaf groups of the three *M. truncatula* natural variants, PI516927, PI516939, and PI577609, also occupied distinct space in the 3D morphospace (Figures 13B–D). Interestingly, the space occupied by the seven leaf groups of four genotypes appeared to be different and consistent with the differences in blade and serration parameters of the leaf groups observed among the genotypes (Figures 10, 11).

### Quantitative analysis and classification of blade and serration features of *M. truncatula* leaf shape mutants

**Figure 14:**
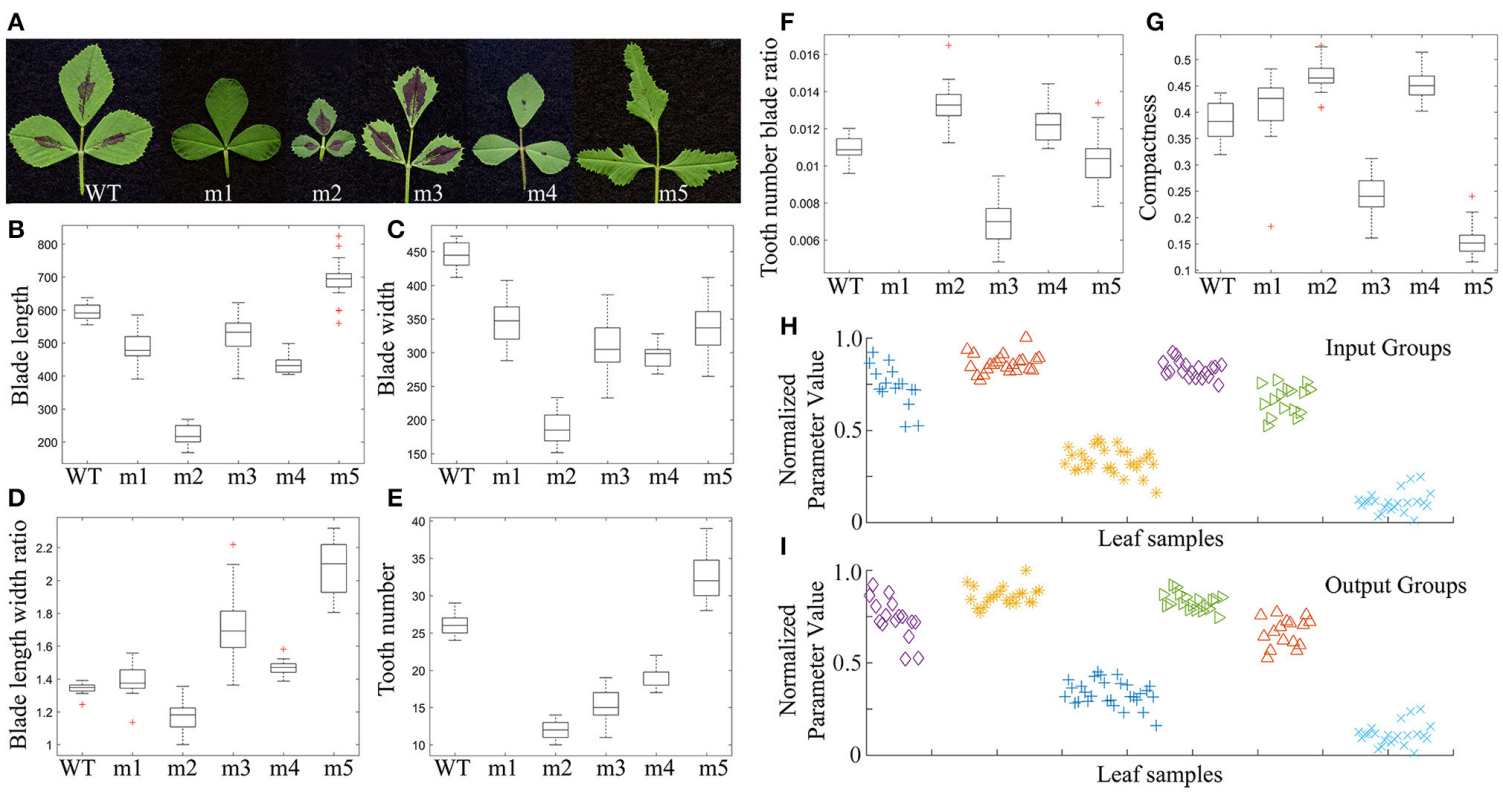
Comparison and classification of leaf shape mutants of *Medicago truncatula*. **(A)** Morphologies of mature leaves of 2-month-old *M. truncatula* wild type (WT; Jemalong A17), and FN3296 (m1), FN1923 (m2), FN20816 (m3), FN1924 (m4), and FN10068 (m5) mutant plants. **(B–G)** Box plots of the leaflet blade and serration parameters, blade length **(B)**, blade width **(C)**, blade length width ratio **(D)**, total tooth number **(E)**, tooth number blade ratio **(F)**, and compactness **(G)**. Middle lines denote the median values, upper and lower edges of boxes denotes the 75th and 25th percentiles, respectively, whiskers denote the most extreme data points that are not considered outliers, and outliers are marked individually, *n* = 9–20. **(H,I)** Classification of leaf sample groups using the *k*-means clustering method. **(H)** Leaf sample groups before classification. **(I)** Leaf sample groups after classification. Shown are leaf sample groups from m1, m2, m3, m4, WT, and m5 plants from left to right.

We measured and compared leaflet blade and serration features of five leaf shape mutants isolated from a collection of *M. truncatula* fast neutron bombardment (FNB)-induced mutants with the corresponding wild type, *M. truncatula* cv. Jemalong A17 (Figure 14A). Nine to 20 replicates of mature trifoliate leaves of 2 months-old plants were used. The results show that leaflets of FN10068 (m5) and FN1923 (m2) had the longest and shortest blade length, respectively (Figure 14B), leaflets of wild type (WT) and FN1923 (m2) had the widest and narrowest blade width, respectively (Figure 14C), leaflets of FN10068 (m5) and FN1923 (m2) had the largest and smallest blade length width ratio, respectively (Figure 14D), and leaflets of FN3296 (m1) had smooth margins (no serrations) and leaflets of FN10068 (m5) and FN1923 (m2) had the largest and smallest number of teeth (Figure 14E). Figure 14F shows that the tooth number blade ratio was zero for FN3296 (m1), but the largest and smallest for leaflets of FN1923 (m2) and FN21806 (m3), respectively. Analysis results further show that the blade compactness was the highest and lowest for leaflets of FN1923 (m2) and FN10068 (m5), respectively (Figure 14G), supporting that leaflets of FN1923 are close to a circle whereas leaflets of FN10068 are preferentially expanding along the proximodistal direction (Figure 14A).

We also tested whether the quantitative results can be used to classify leaflet groups in the mutants. All of the leaflet samples from mutants and wild type plants were first mixed and then regrouped using the *k*-means clustering method with a combination of three or more blade and serration parameters optimized by the genetic algorithm (Figure 4). Supplementary Table 4 lists ten combinations of parameters that were used in the classification. The results show that the classification accuracy ranged from 96 to 100% (Supplementary Table 4; Figures 14H,I).

## Discussion

Analyzing leaflet blade and margin features by hands or by software such as ImageJ that requires manual measurement is extremely time consuming, making it impossible to quantitatively analyze a large number of leaflet samples. In this study, we developed the software, LeafletAnalyzer, for automated image processing, measurement and calculation of 54 leaflet size, shape and tooth features, statistical analysis and classification of compound leaves of *M. truncatula* plants, a model legume species closely related to many economically important crops including alfalfa and soybean.

In contrast to previously-reported software, which measures parameters for a few reference points manually selected on leaf images, or uses the peak and valley positions of leaf teeth or triangles as proxies for leaf teeth, LeafletAnalyzer detects leaflet borders and measures parameters for all position points (pixels) on leaflet borders. Therefore, this software can precisely calculate leaflet shape and size and parameters for serrations and sinuses at the leaflet margin. Leaf serrations (teeth) are important features that can be seen in many plant species but have not been characterized quantitatively due to lack of software designed to detect, measure and analyze serration features. Numberous studies have shown that leaf teeth are associated with the mean annual temperature (MAT) and local water availability (Royer and Wilf, 2006; Peppe et al., 2011). The percentage of woody, eudicot species with toothed leaves and variables related to tooth count and size all negatively correlate with MAT (Royer and Wilf, 2006; Peppe et al., 2011). It is postulated that the prevalence of leaf teeth in cool climates is an adaptation for increased carbon uptake through enhanced sap flow early in the growing season (Royer and Wilf, 2006; Peppe et al., 2011). In warmer climates, the potential benefit of an increased carbon uptake is outweighed by the associated water loss (Royer and Wilf, 2006; Peppe et al., 2011). Leaf size is also sensitive to climate, particularly local water availability and to a lesser degree temperature. Plants in drier environments tend to have smaller leaves to reduce evaporative cooling, while in more humid environments larger leaves are common. However, most of the paleoclimate studies rely heavily on labor-intensive manual measurements of leaf samples.

Because of the automation feature, LeafletAnalyzer can measure, analyze and classify thousands of leaf samples at a time. In addition to the automated version, LeafletAnalyzer includes a manual version to allow users to pause the automatic process and to make manual adjustments, such as manually selecting the blade length and tooth positions. The blade length is typically the length of the mid-vein of leaflets. In wild-type and most mutant and ecotype plants, the midvein of leaflets usually straight and the automatic selection by the software is mostly accurate. However, in some leaf shape mutants (Figure 2G), in which the midvein of a leaflet is curved, the software would not accurately select the midvein of the leaflet. In this case, the leaflet length will have to be selected manually. On the other hand, some leaflet images have broken points on leaflet margins. These broken points may be selected as teeth by the software, although the software has algorithm to reduce the possibility. However, if error detection occurs, users can use the manual version to correct it. Once manual adjustments are made, the software will resume the automated process of measurements and analysis.

In *M. truncatula*, leaves developed on successive nodes of the stem appear different in sizes and shapes, a phenomenon known as heteroblasty (Wang et al., 2008; Peng et al., 2011; Ge et al., 2014). However, quantitative analysis of leaf development in *M. truncatula* has not been described before. Our analysis of leaflet blade and margin features shows that L2 and L3 in 6 weeks-old wild type plants have the largest leaflet area and the leaflet area is sharply increased from L1 to L2 and decreased from L3 to L6. These are attributed more by an increased and decreased development along the proximodistal axis than the mediolateral axis as supported by an increase and decrease in the blade length width ratio from L1 to L2 and L2 to L6, respectively (Figure 5).

The total tooth number is the highest in L1 but decreased from L1 to L6 (Figure 6A). Interestingly, the total tooth area is the highest in L2 and decreased from L2 to L6 (Figure 6A). On the other hand, the average tooth area and the tooth valley ratio are increased from L2/L3 to L6, indicating that teeth on average are getting bigger from L3 to L6, although the total tooth area is decreased due to reduced tooth number (Figure 6). These results indicate that a big leaflet such as L2 or L3 has a large total tooth area, but not necessarily a high total tooth number or average tooth area (Figure 6). The average tooth tip angle is increased from L1 to L4 but decreased from L4 to L6, indicating that the shape of teeth also changes from L1 to L6. The quantitative results are consistent with previous observations that leaves developed during early plant growth have smooth margin and are smaller and rounder than those developed at later stages h (Wang et al., 2008; Peng et al., 2011; Ge et al., 2014).

It has been reported that plants with serrated leaf margin are more active in photosynthesis and transpiration than leaves with smooth margin (Royer et al., 2008). Our observation that leaf features change during leaf development may reflect the adaptive nature of leaf development during seedling establishment, which may require less active photosynthesis and transpiration.

Using LeafletAnalyzer, we measured and analyzed leaflet blade and margin features of five *M. truncatula* leaf shape mutants. Our analysis shows that, compared with wild type leaflet samples, all mutant leaflets exhibited reduced blade width, suggesting that each underlying genes responsible for the corresponding mutant leaf phenotypes may play a role in mediating the mediolateral expansion of leaflet blades during leaf development. On the other hand, FN10068 (m5) and the other four mutants had longer and shorter blades, respectively, than wild type. This may suggest that the underlying genes in FN10068 (m5) and the other mutants may play different roles in the proximodistal expansion of leaflet blades.

FN1923 (m2) had the lowest values of the blade length and width but the highest value of the leaflet compactness(Figure 14G), indicating that its leaflet blades are the smallest and close to a circle (Figure 14A). By contrast, FN10068 (m5) and FN20816 (m3) had smaller compactness values and higher blade length width ratios than others, indicating that their blades are far from a circle (Figure 14G).

Analysis of serration features shows that FN10068 (m5) had the highest tooth number and FN3296 had no teeth (Figure 14E). Interestingly, although FN1923 had the second smallest tooth number, it had the highest tooth number blade ratio because of its extremely small blade area (Figure 14F).

We used the *k*-means clustering method to classify leaf groups of these mutants and wild type. Supplemental Table 4 lists ten combinations of blade and serration parameters that are used to classify leaf groups among the mutants and wild type at an accuracy rate between 96 and 100%. The classification accuracy is higher than those for leaf groups in wild type plants that were calculated based on an artificial neural network. By contrast, the classification accuracy was low for leaf groups in wild type if calculated based on the *k*-means clustering method (data not shown), suggesting that the *k*-means clustering method can be used to classify accurately leaf groups in genotypes with distinct blade and serration features, whereas an artificial neutral network method can be used to classify accurately leaf groups in genotypes with less distinct leaf features.

LeafletAnalyzer was also used to measure, analyze and compare leaflet blade and serration features of three randomly selected *M. truncatula* natural variants, PI516927, PI516939, and PI577609 and the reference plant, *M. truncatula* cv. Jemalong A17 (Figure 9). Our analysis results show that, within each genotype, the seven leaflet groups (L1 to L7) of 45 days-old plants can be classified at above 90% accuracy (Supplementary Table 3). On the other hand, the four genotypes can be classified using each leaflet groups at above 90% accuracy (Supplementary Table 4), indicating that leaflet blade and serration features differ significantly among the four genotypes.

Interestingly, the relationship between blade and serration features differs in these genotypes. For example, both PI516939 and A17 had smaller blade area and lower tooth number than the other two genotypes in several leaflet groups (Figures 10A, 11A). But, A17 and PI516939 had the highest and lowest relative tooth area, respectively, among the four genotypes (Figure 11C). On the other hand, although both PI516927 and PI577609 had higher tooth number and larger total tooth area than the other two genotypes, the relative tooth area was intermediate between A17 and PI516939 (Figure 11C).

In summary, we have developed LeafletAnalyzer to measure and analyze leaflet morphologies and dynamic changes during compound leaf development in *M. truncatula*. We show that LeafletAnalyzer can automatically process a large number of leaf samples, measure blade and margin features and classify leaflet groups using the *k*-means clustering and artificial neural network methods. With this software, we are able to identify dynamic changes in blade and margin features during compound leaf development in wild type plants and compare them among natural variants (ecotypes) and developmental mutants. We have also used this software in quantitative analysis of leaves of alfalfa (*M. sativa*) and tomato (*Solanum lycopersicum*) with slight modification of processes related to trichome removal and leaf margin feature calculation. Because of the quantitative nature, leaflet blade and margin features generated by LeafletAnalyzer may have multiple potential applications in areas such as plant taxonomy, paleoclimate studies, mutant characterization, quantitative trait loci (QTL) and genome wide association mapping (GWAS) of leaf traits.

## Author contributions

RC planned and designed the research; FL wrote the computer codes; JP performed experiments; FL, JP, and RC analyzed the data; RC, FL, JP wrote the manuscript.

### Conflict of interest statement

The authors declare that the research was conducted in the absence of any commercial or financial relationships that could be construed as a potential conflict of interest. The reviewer DM and handling Editor declared their shared affiliation, and the handling Editor states that the process met the standards of a fair and objective review.
