# Supplementary material for: LeafletAnalyzer, an Automated Software for Quantifying, Comparing and Classifying Blade and Serration Features of Compound Leaves during Development, and among Induced Mutants and Natural Variants in the Legume Medicago truncatula
Source: Front Plant Sci. 2017 May 31;8:915. doi: 10.3389/fpls.2017.00915 (PMC5450422; doi:10.3389/fpls.2017.00915)
Supplement: Supplementary file 5 [file DataSheet1.docx]

**Supplementary Materials**

**Supplementary Figure S1.** Automated separation of terminal and lateral leaflets from petiole/rachis by the software. (**A)** A plot of distances between the centroid and each point on the leaflet margin and angles between the centroid and each point on the leaflet margin relative to the horizontal line. For two lateral leaflets, four turning points as marked by colored dots denote the left and right borders of leaflet and petiole junctions. (**B)** An outline of a compound leaf. Colored lines represent different sections of the leaf margin. Lateral leaflets were separated at the left and right borders of the junction from the petiole. (**C)** The original leaf image.

**Supplemental Table S1.** Ten combinations of three leaflet blade and serration parameters used in the classification of six leaf groups in six-week-old wild-type *Medicago truncatula* plants. The classification results were compared with the original leaf groups to calculate the classification accuracy.

**Supplementary Table S2.** Ten combinations of three leaflet blade and serration parameters used in the classification of three *Medicago truncatula* natural variants, PI516927, PI516939 and PI577609 and the reference plant *M. truncatula* cv. Jemalong A17, based on individual leaflet groups. The classification results were compared with the original leaf groups to calculate the classification accuracy.

**Supplementary Table S3.** Ten combinations of leaflet blade and serration parameters used in the classification of seven leaflet groups in three *Medicago truncatula* natural variants, PI516927, PI516939 and PI577609 and the reference plant *M. truncatula* cv. Jemalong A17. The classification results were compared with the original leaf groups to calculate the classification accuracy.

**Supplementary Table S4.** Ten combinations of three or more blade and serration parameters used in the classification of *Medicago truncatula* leaf shape mutants and wild type. The classification results were compared with the original leaf groups to calculate the classification accuracy.

**Supplementary File 1.** Raw data of compound leaves during leaf development in *M. truncatula* wild type plants.

**Supplementary File 2.** Raw data of compound leaves in *M. truncatula* natural variants and the reference plant.

**Supplementary File 3.** Raw data of compound leaves in *M. truncatula* leaf developmental mutant and wild type plants.

**Supplementary File 4.** Screenshots of the operational steps of LeafletAnalyzer.

Screenshot 1, Open Matlab and create a folder such as “Demo” to host original leaf images such as 5-1 to 9-1;

Screenshot 2, Input the software name ‘leafz.m’ in Matlab;

Screenshot 3, Run ‘leafz.m’ and the LeafletAnalyzer interface appears on the screen; Left click ‘Medicago’ button on the LeafletAnalyzer interface;

Screenshot 4, LeafletAnalyzer opens a dialog box and lets user select the folder of leaf images to be analyzed, in this case, “Demo”. Left click ‘OK’. LeafletAnalyzer will begin to automatically process leaf images one by one in the folder. While the software is running, the ‘Medicago’ button stays blue. When the software finishes, it changes to red.
