# Supplementary material for: LeafletAnalyzer, an Automated Software for Quantifying, Comparing and Classifying Blade and Serration Features of Compound Leaves during Development, and among Induced Mutants and Natural Variants in the Legume Medicago truncatula
Source: Front Plant Sci. 2017 May 31;8:915. doi: 10.3389/fpls.2017.00915 (PMC5450422; doi:10.3389/fpls.2017.00915)
Supplement: Supplementary file 9 [file File4.PDF]

Step 1: Open ‘MATLAB’.

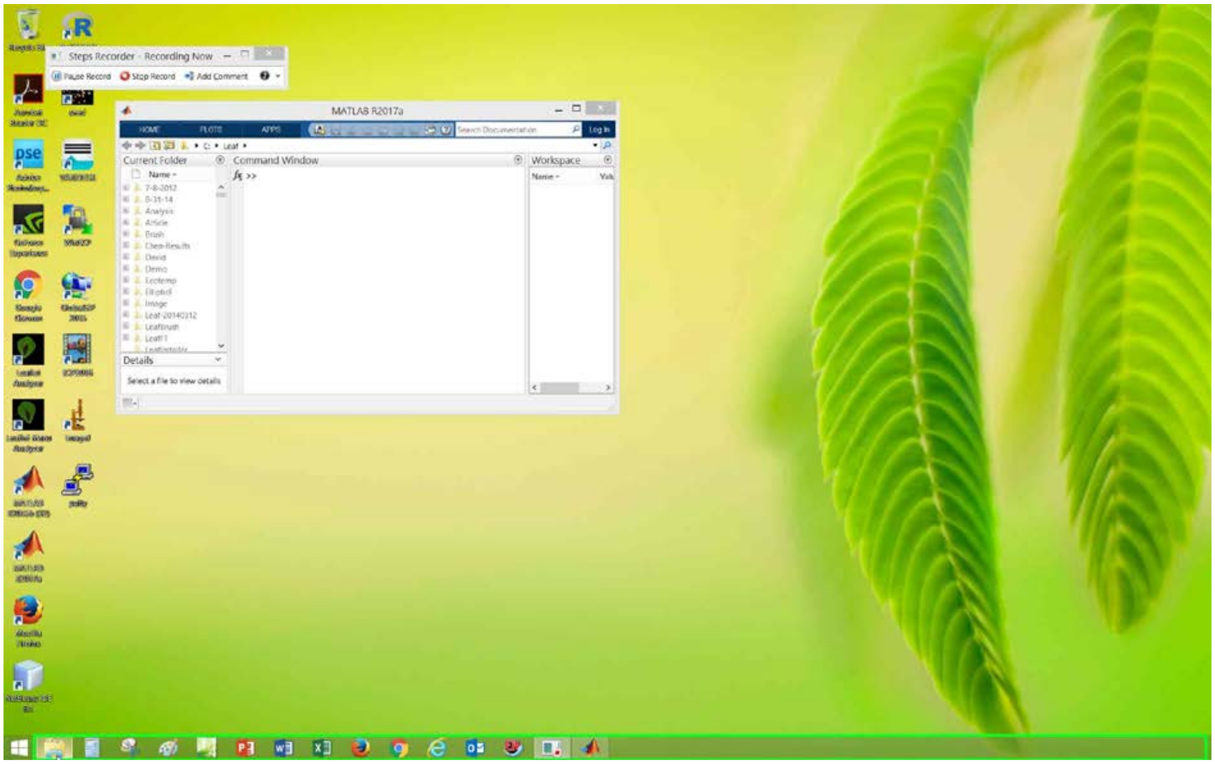

Step 2: Open "Demo" folder, in which five leaf images are to be analyzed by LeafletAnalyzer.

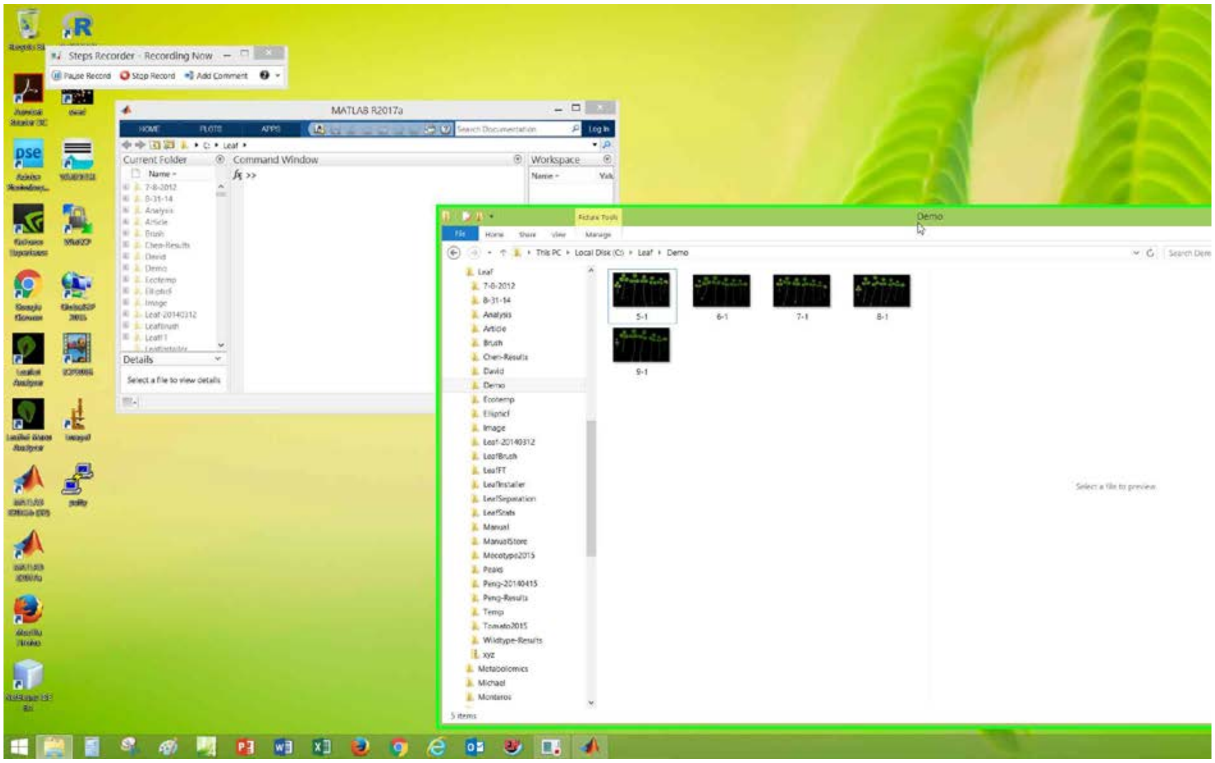

Step 3: In MATLAB, enter the name of the software ‘leafz.m’

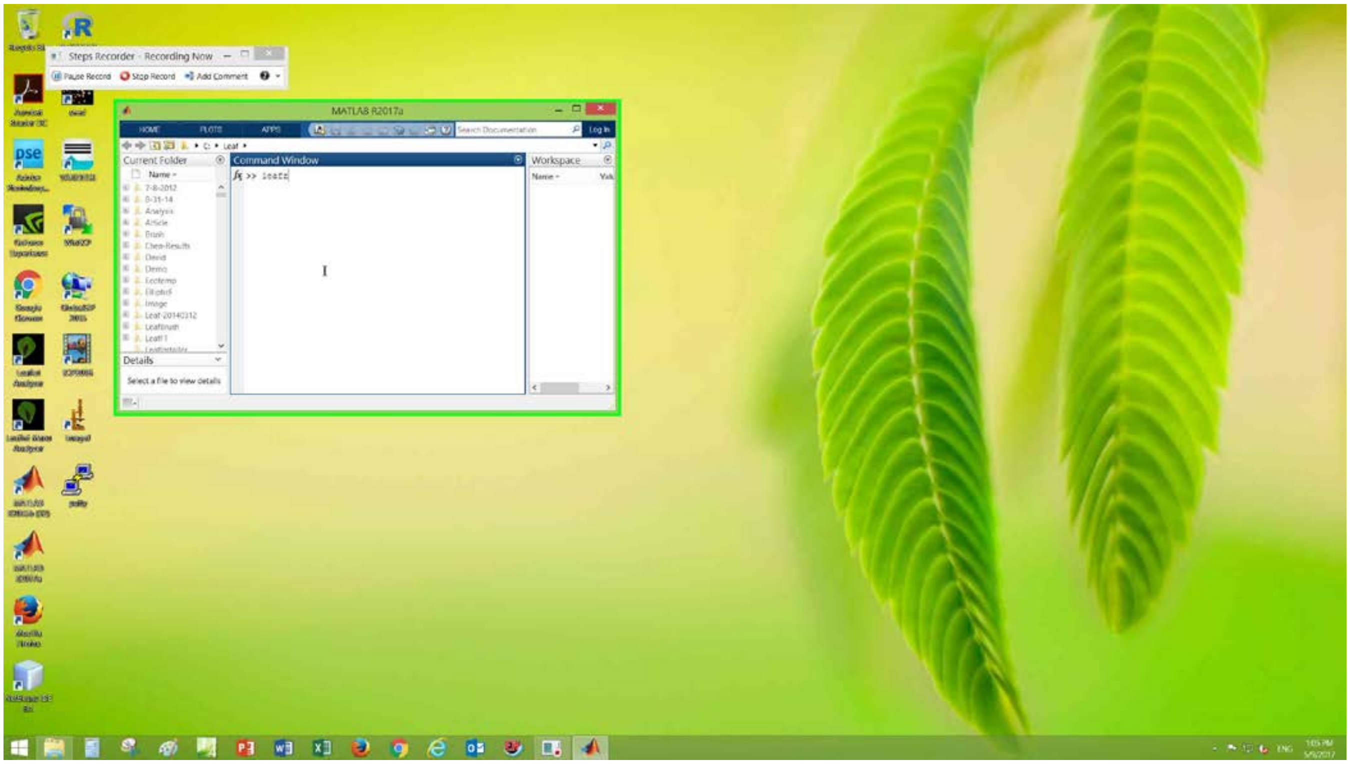

Step 4: The interface of ‘LeafletAnalyzer’ appears.

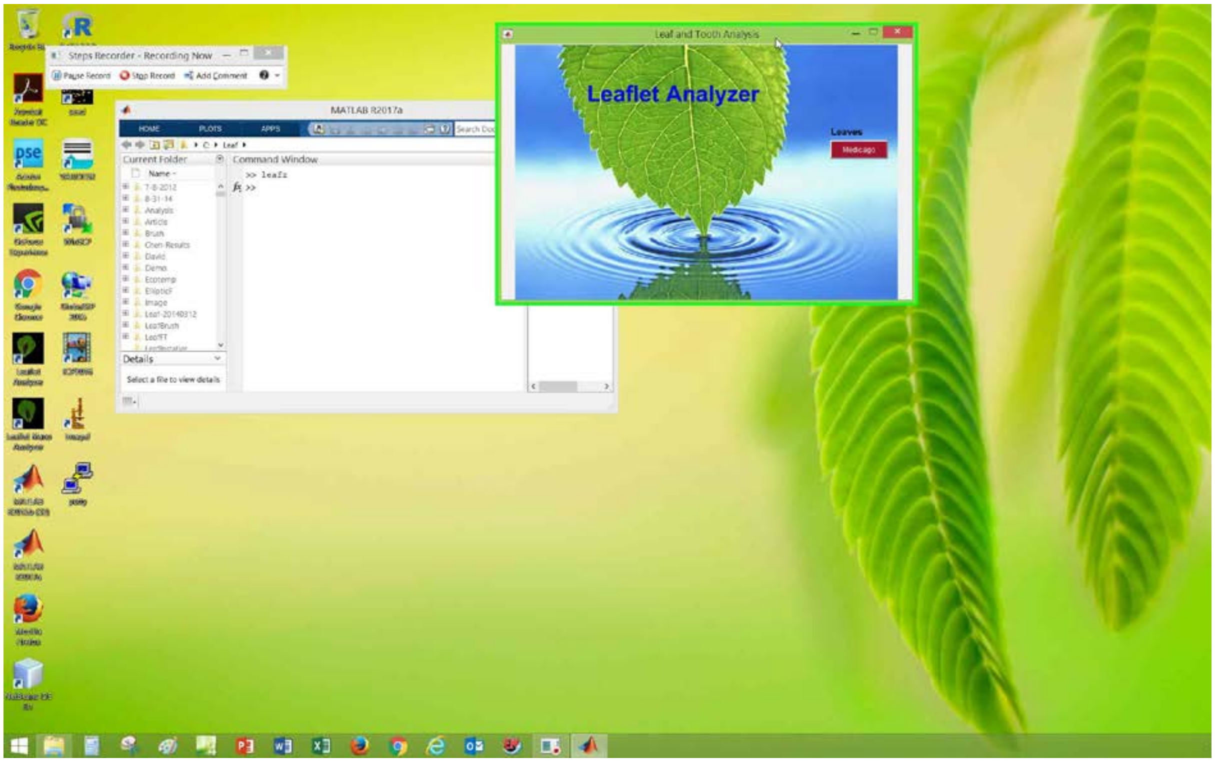

Step 5: Click the ‘Medicago’ button on the interface.

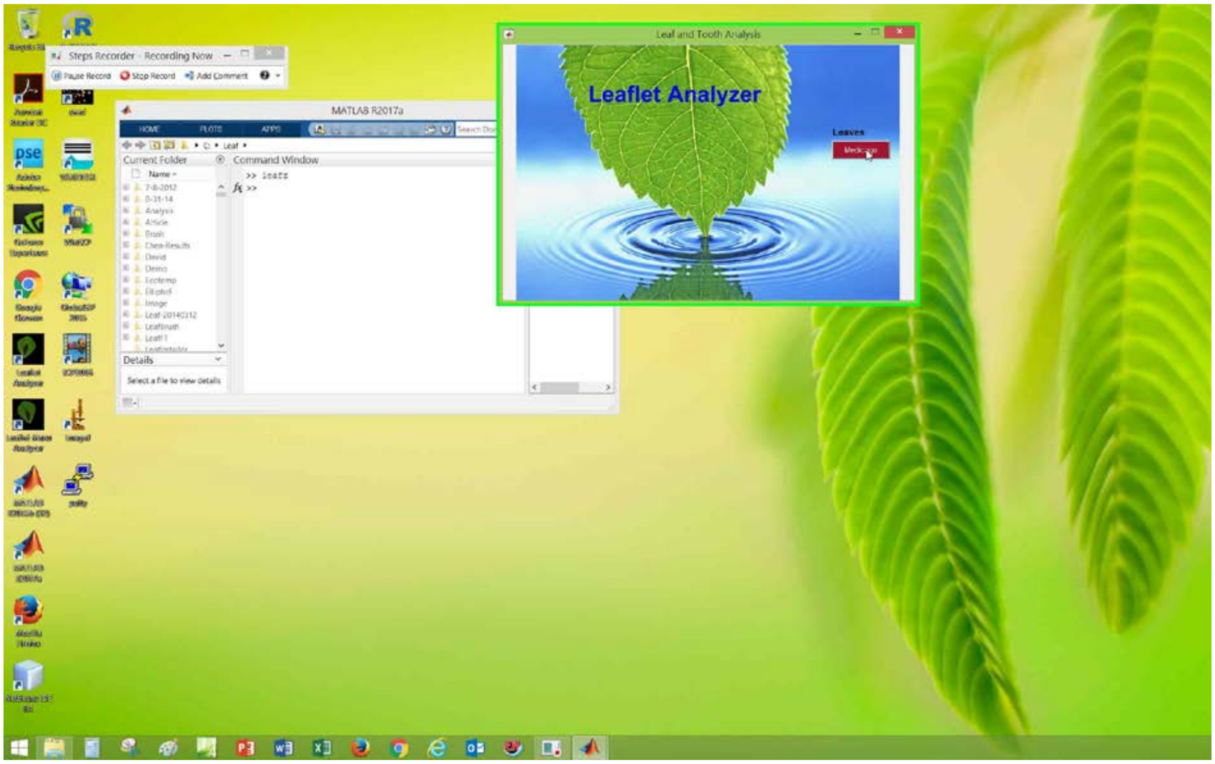

Step 6: A dialog box is open and asks user to select the folder of leaf images.

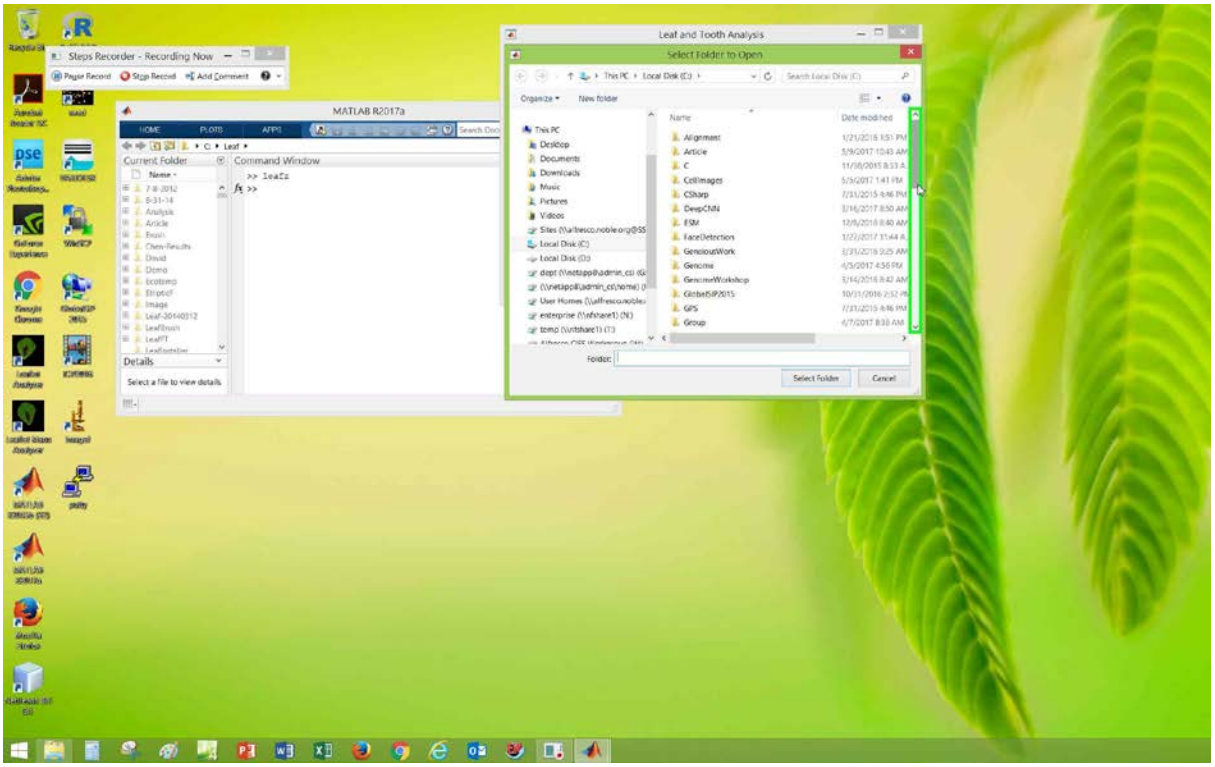

Step 7: The ‘Demo’ folder is selected.

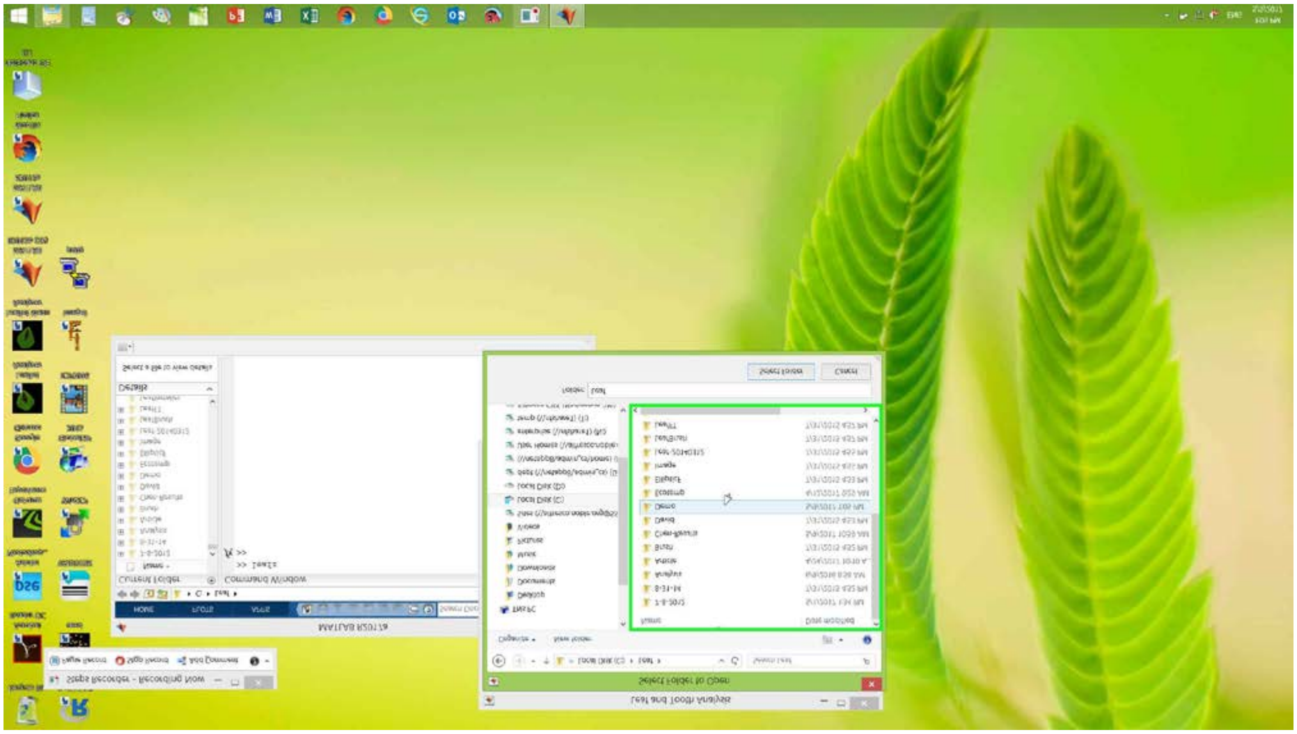

Step 8: After the image folder is selected, the ‘Medicago’ button will be in blue color. LeafletAnalyzer will automatically begin working on image processing for each leaf images.

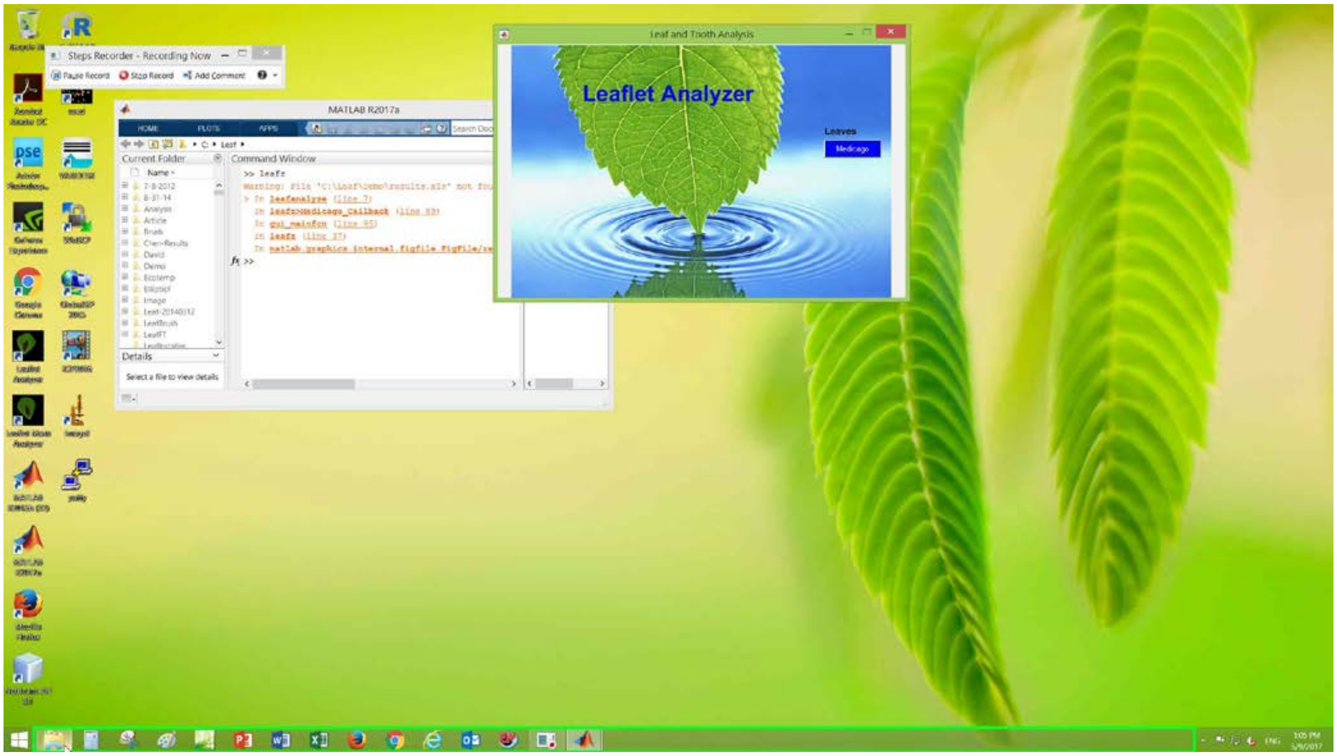

Step 9: After LeafletAnalyzer finishes processing of all leaf images, the 'Medicago' button will return to red color. The following screenshots show that processed graphs for each input leaf images will be generated by the software and stored in the same folder 'Demo'. These processed graphs show how LeafletAnalyzer works. User can see these graphs during the operation of LeafletAnalyzer.

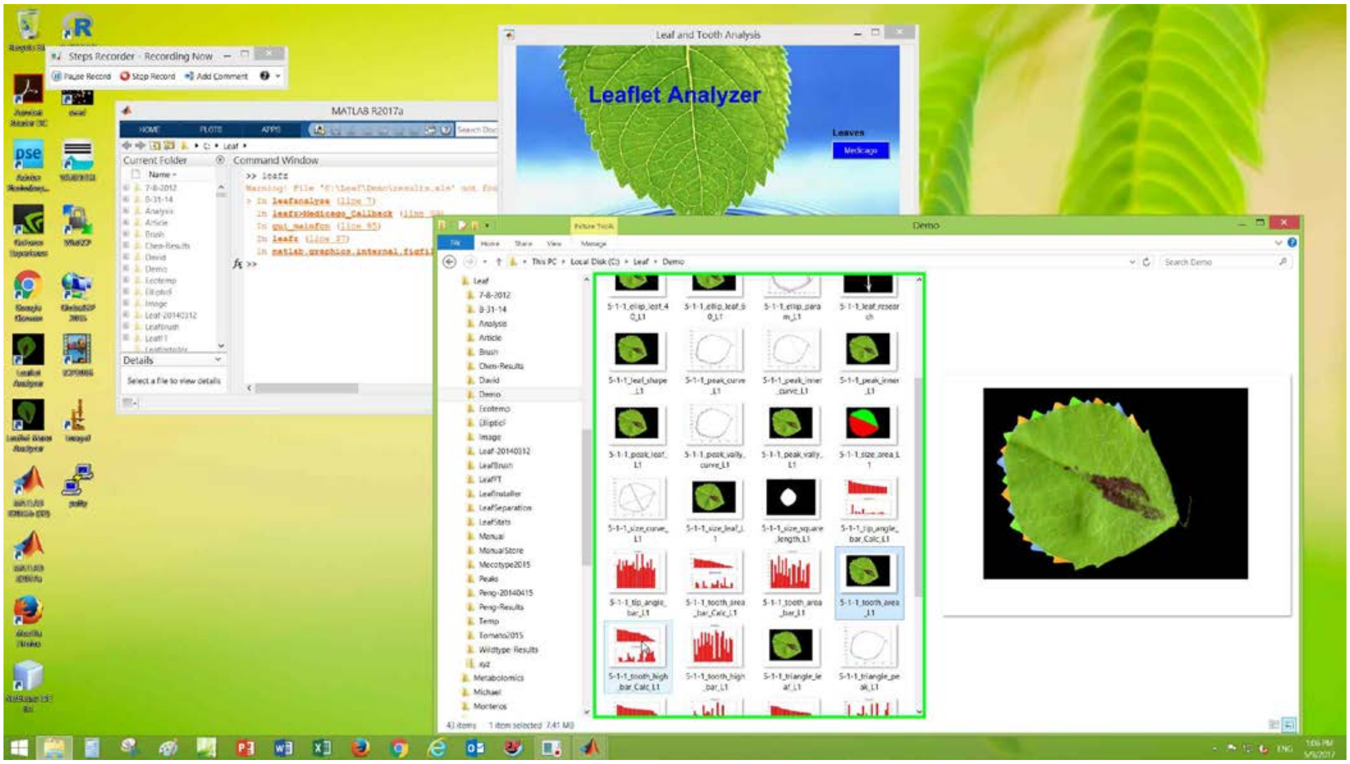
Steps Recorder - Recording Now
Pause Record
Stop Record
Add Comment
MATLAB R2017a
Current Folder
Command Window
Leaf and Tooth Analysis
Leaflet Analyzer
Leaves
Medicago
Demo

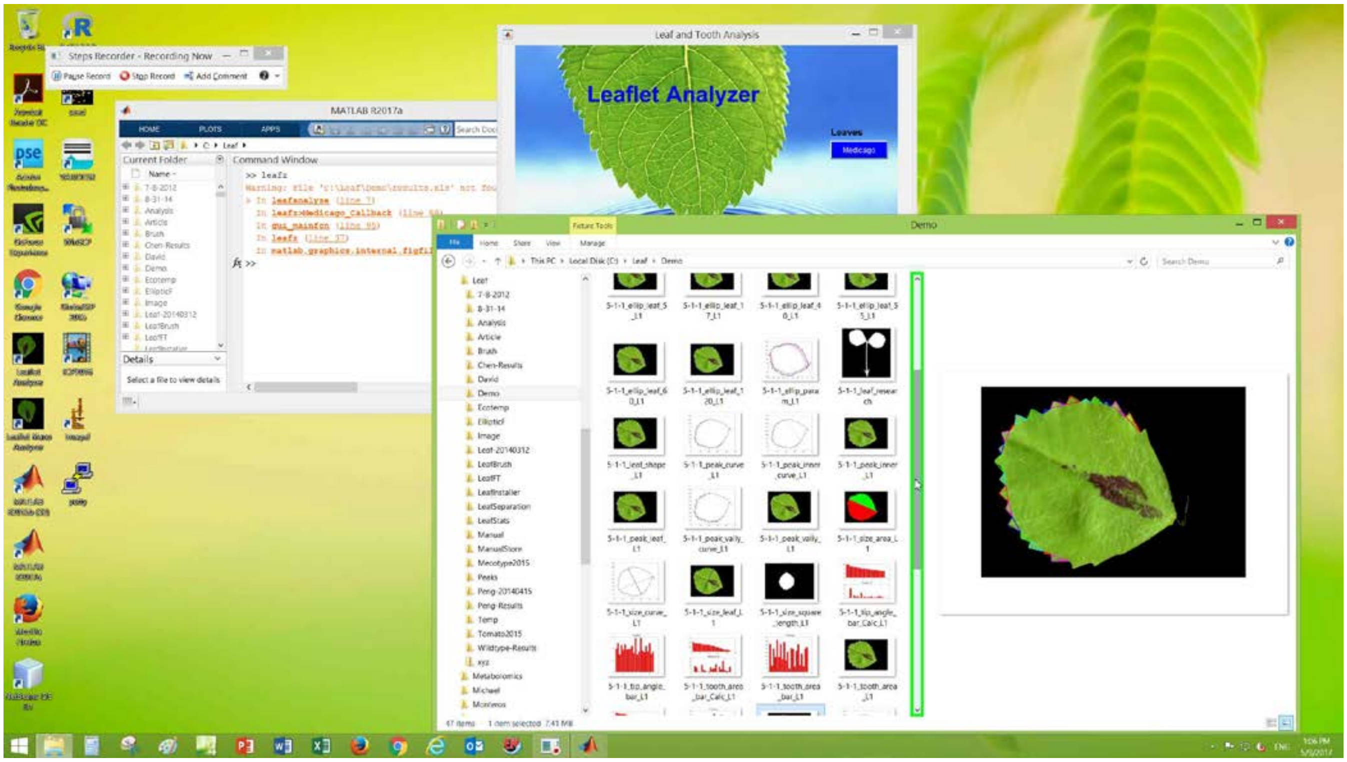
Steps Recorder - Recording Now
Pause Record
Stop Record
Add Comment
MATLAB R2017a
Current Folder
Command Window
Leaf and Tooth Analysis
Leaflet Analyzer
Leaves
Medicago
Demo

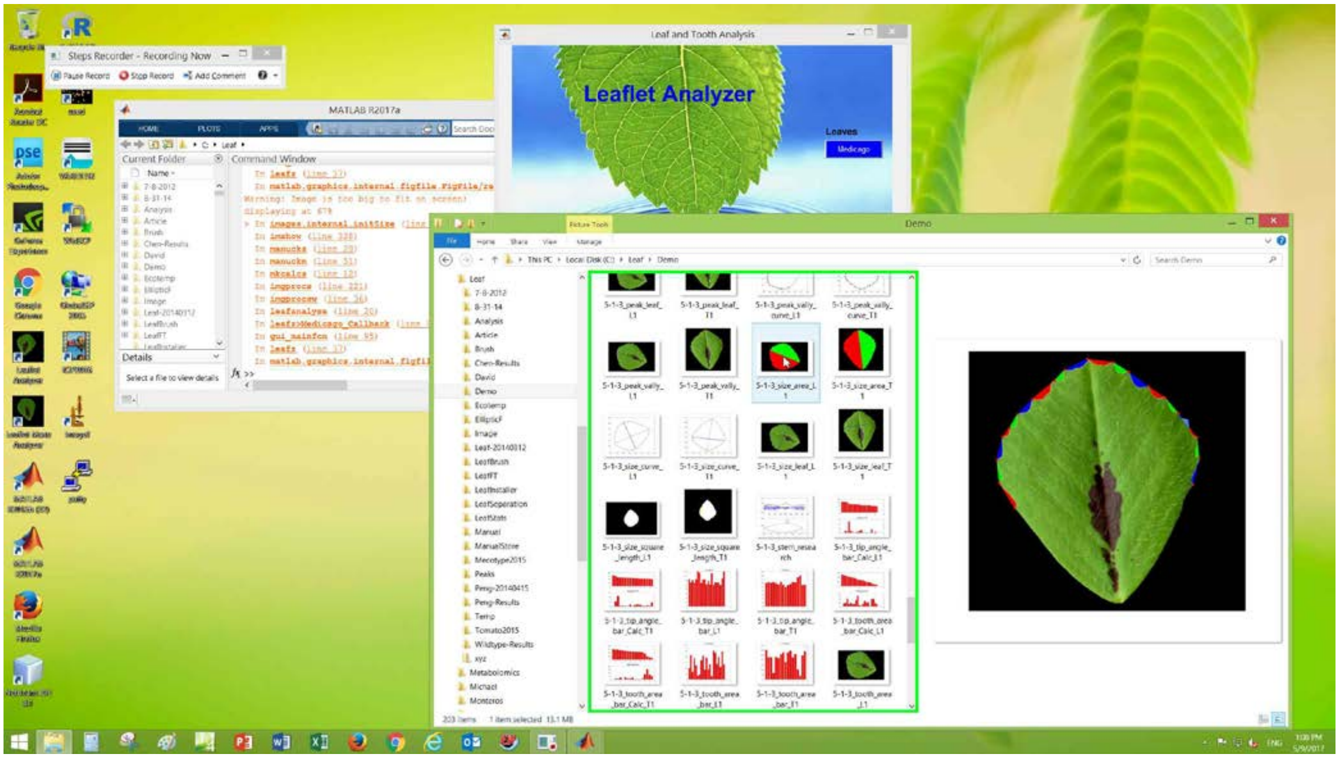
Steps Recorder - Recording Now
MATLAB R2017a
Leaf and Tooth Analysis
Leaflet Analyzer
Demo

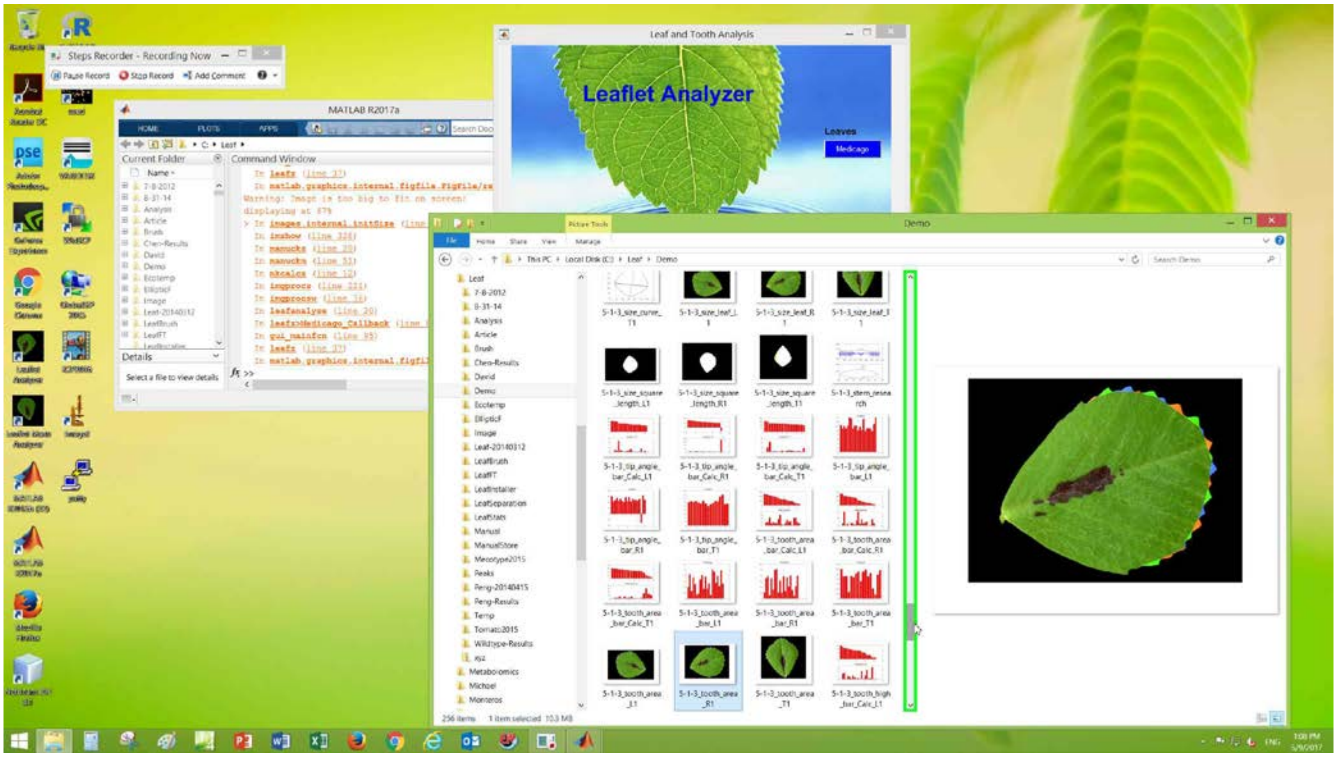
Steps Recorder - Recording Now
MATLAB R2017a
Leaf and Tooth Analysis
Leaflet Analyzer
Demo
