# Supplementary figures and images for: LeafletAnalyzer, an Automated Software for Quantifying, Comparing and Classifying Blade and Serration Features of Compound Leaves during Development, and among Induced Mutants and Natural Variants in the Legume Medicago truncatula
Source: Front Plant Sci. 2017 May 31;8:915. doi: 10.3389/fpls.2017.00915 (PMC5450422; doi:10.3389/fpls.2017.00915)

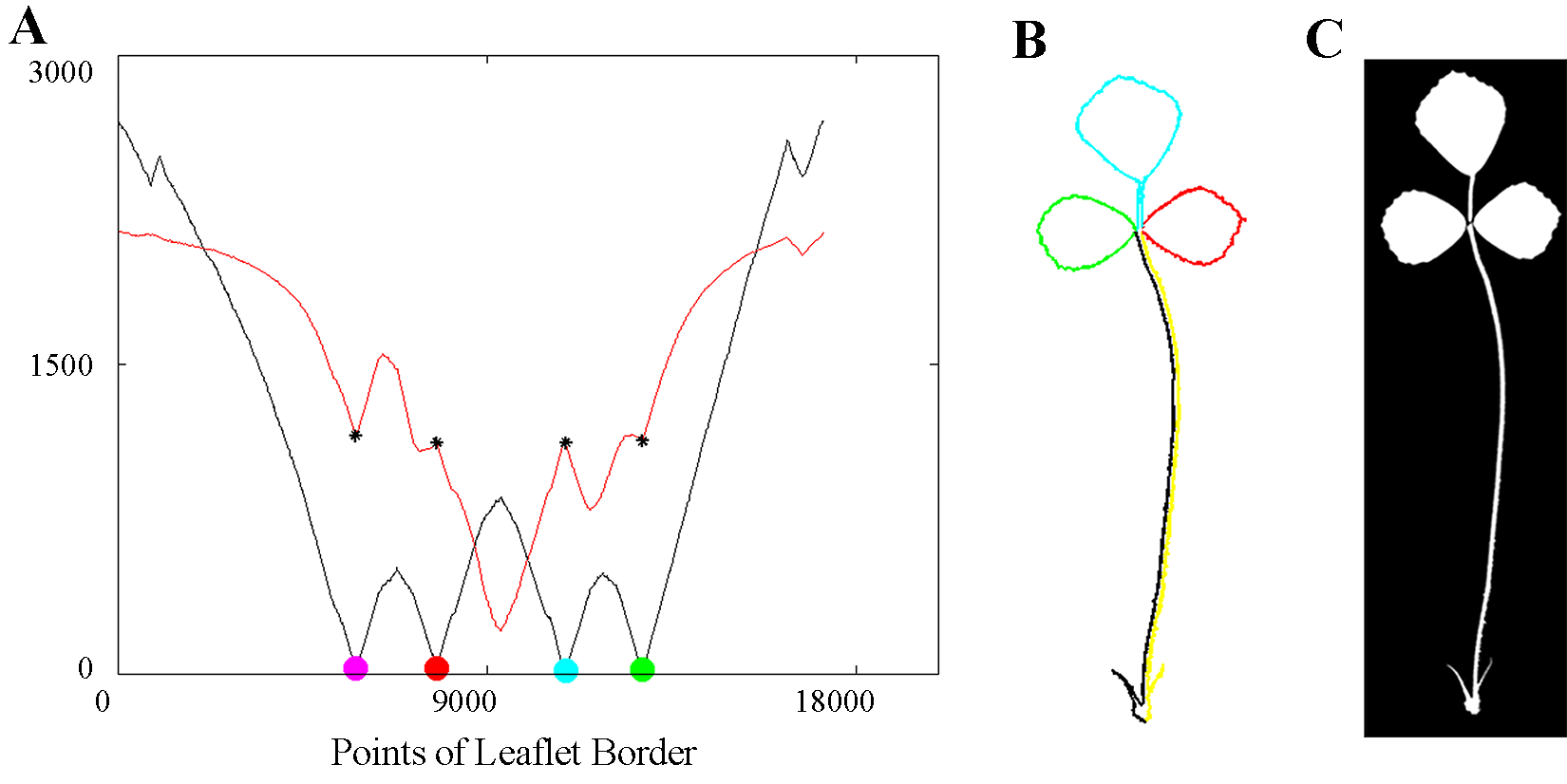

Supplement: Supplementary file 10 [file Image1.TIF]
